# Benchmarking five large language models in medical genetics: a bilingual comparative evaluation using published and novel expert-authored questions

**DOI:** 10.3389/fmed.2026.1938886

**Published:** 2026-09-15

**Authors:** Özge Beyza Gündoğdu Öğütlü, Benjamin D. Solomon, Yusuf Selman Çelik

**Affiliations:** 1 Department of Medical Genetics, Ankara Etlik City Hospital, Ankara, Türkiye; 2 Medical Genomics Unit, National Human Genome Research Institute, National Institutes of Health, Bethesda, MD, United States; 3 Department of Child and Adolescent Psychiatry, Kırıkkale University, Kırıkkale, Türkiye

**Keywords:** artificial intelligence, educational measurement, genetics, medical, large language models, multilingualism

## Abstract

This study asked whether five contemporary large language models answer medical genetics multiple-choice questions with equivalent accuracy on published versus novel items and across English and Turkish, and sought to characterize the errors that persist. Five models (GPT-5.2, Gemini 3 Pro, Claude Sonnet 4.6, Grok 4, and DeepSeek-V3.2) answered 100 four-option questions (50 from a published board review; 50 novel, expert-authored items absent from any database) in English and Turkish, yielding 1,000 responses. Correctness was modeled with item-clustered generalized estimating equation and Bayesian mixed-effects logistic regression (the latter as a prespecified sensitivity analysis); question provenance and language were tested for equivalence (item-clustered two one-sided tests, ±5-percentage-point margin), and the paired language effect with the McNemar test. Inter-model agreement and error concordance were examined. Overall accuracy was 97.7%. In the item-clustered GEE, only Gemini 3 Pro nominally exceeded the lowest-performing model; this imprecise contrast did not remain significant after Holm correction for the four secondary model comparisons, whereas the Bayesian sensitivity analysis additionally yielded a credible interval excluding 1 for GPT-5.2 versus Grok 4. Accuracy was statistically equivalent within the prespecified margin for published versus novel items (difference, −1.4 percentage points; item-clustered 90% CI, −4.1 to +1.3; item-clustered TOST *P* = 0.014) and for English versus Turkish (difference, −0.6 points; paired item-level TOST *P* < 0.001; McNemar *P* = 0.58). Inter-model agreement on the exact option chosen was high (Fleiss *κ*, 0.94–0.97). All five items with two or more errors failed concordantly, every erring model selecting the same wrong option. Beyond high accuracy, distinct model families converged on identical answers and, on the hardest items, on identical errors, a pattern consistent with shared learned associations and/or overlapping training data, although this behavioral comparison cannot identify the underlying mechanism. In an exploratory analysis, residual errors showed concentrated and concordant patterns, clustering in multistep Bayesian reasoning and evolving facts, suggesting that a second model may provide limited independent protection on such items. Performance on this restricted multiple-choice benchmark does not establish readiness for clinical genetic counseling, variant interpretation, or quantitative risk assessment, and human oversight remains essential.

## 1 Introduction

The integration of artificial intelligence (AI) into medical education and clinical practice has accelerated remarkably, with large language models emerging as tools capable of synthesizing complex biomedical information with near-human proficiency. Recent systematic reviews and meta-analyses have demonstrated that contemporary large language models achieve passing or expert-level scores on standardized medical licensing examinations across multiple disciplines (1–3). In particular, Singhal et al. reported that Med-PaLM 2 exceeded the passing threshold on United States Medical Licensing Examination (USMLE)-style questions (4), while subsequent benchmarking of GPT-5 demonstrated 94.1% accuracy on the MedQA dataset (5). These advances have prompted calls for domain-specific evaluations to determine which medical specialties benefit most from AI augmentation and where critical limitations persist (6, 7).

Medical genetics occupies a distinctive position in this landscape. Unlike disciplines that rely primarily on subjective clinical judgment or visual pattern interpretation, genetics encompasses a substantial body of codified, rule-governed knowledge (inheritance patterns, molecular mechanisms, syndrome–phenotype associations, and quantitative risk assessment) that is, in principle, well-suited to the pattern-matching and retrieval strengths of large language models. At the same time, genetics involves complex probabilistic reasoning (e.g., Bayesian carrier risk calculation and Hardy–Weinberg equilibrium estimation) and rapidly evolving factual knowledge, which may expose specific weaknesses in current large language model architectures (8, 9). The foundational study by Duong and Solomon provided the first systematic assessment of large language model performance on genetics questions, demonstrating that ChatGPT (GPT-3.5) achieved 68.2% accuracy, comparable to human respondents, but performed significantly better on memorization-type items than on those requiring critical thinking (10). Flaharty et al. subsequently extended this line of inquiry by systematically evaluating open- and closed-source architectures across clinician-authored and layperson-translated descriptions of 63 genetic conditions, demonstrating that both model scale and input formulation exert differential effects on identification accuracy (11). Subsequent evaluations confirmed that GPT-4 showed improved accuracy but continued to struggle with nuanced clinical scenarios (12, 13).

A persistent and methodologically important critique in current medical AI benchmarking is the “data contamination” concern: whether high accuracy on established question banks reflects genuine reasoning or memorization of training data that may include published test items (14, 15). This concern is particularly relevant for genetics, where canonical textbook questions [such as those from the established board review question bank (16)] may plausibly appear in pre-training corpora. To date, no study has directly addressed this issue by comparing large-language-model performance on published versus entirely novel unpublished genetics items within a single experimental framework.

Beyond accuracy *per se*, the cross-linguistic dimension of large language model performance in genetics remains unexplored. Prior studies in other medical domains have reported mixed findings: ChatGPT-4 achieved 91% accuracy on the French ophthalmology board examination (17) but only 73.6% on the Chinese National Medical Licensing Examination (18), suggesting that language and cultural context may influence performance. More recent large-scale analyses, including a 4,917-case evaluation of rare disease diagnosis across ten languages, have indicated that contemporary models maintain consistent accuracy across linguistic contexts (19); however, this has not been confirmed for genetics-specific content.

Against this background, this study was designed to address three underexplored and interconnected questions in a single experimental framework: (1) Do contemporary large language models achieve comparable accuracy on published versus novel, unpublished genetics items—thereby providing an exploratory, indirect probe of the data contamination hypothesis, recognizing that near-ceiling accuracy constrains the sensitivity of this comparison? (2) Does the language of administration (English versus Turkish) influence performance on structured genetics knowledge assessments? (3) What specific cognitive demands, identified through systematic item-level error analysis, remain challenging for current-generation models? To answer these questions, five contemporary large language models (GPT-5.2, Gemini 3 Pro, Claude Sonnet 4.6, Grok 4, and DeepSeek-V3.2) were evaluated across 1,000 model–language–item combinations using both published reference questions and novel expert-authored items administered in two languages. The equivalence analyses of question provenance and language were prespecified as the primary analyses; between-model comparisons were secondary analyses corrected for multiplicity, and the agreement and error-concordance analyses were exploratory. This design enables a focused, bilingual benchmark of large language model capabilities in medical genetics while simultaneously disentangling the contributions of question provenance, linguistic context, and cognitive complexity to model performance.

## 2 Materials and methods

### 2.1 Study design and model selection

This cross-sectional comparative benchmarking study evaluated five publicly available large-language models: GPT-5.2 (OpenAI), Gemini 3 Pro (Google), Claude Sonnet 4.6 (Anthropic), Grok 4 (xAI), and DeepSeek-V3.2 (Hangzhou DeepSeek AI Basic Technology Research Co.). These models were selected to represent the major contemporary large-language-model families, encompassing both commercial and open-source architectures. These five systems were recent, widely used releases from five major model families at the time of testing; the designation “contemporary” is therefore anchored to the testing window stated in Section 2.3, where the access details for each model are specified. The study followed a 5 (model) × 2 (question source) × 2 (language) factorial design, yielding 200 responses per model and 1,000 total responses.

### 2.2 Question instrument

The question pool comprised 100 medical genetics multiple-choice questions drawn from two sources to enable a direct comparison between published and novel items:

**Published reference questions (*n*** **=** **50):** Extracted from *Medical Genetics and Genomics: Questions for Board Review* by Solomon (16), a widely used educational resource in genetics training. These items covered inheritance patterns, molecular genetics, cytogenetics, clinical genetics syndromes, and risk calculations.

**Novel expert-authored questions (*n*** **=** **50):** Developed by study investigators through a structured consensus process. Each item was designed to reflect the cognitive demands of genetics board examinations, encompassing clinical vignette-based diagnostic reasoning, syndrome recognition, molecular mechanism identification, and quantitative risk assessment. To reduce author-related bias, questions were developed without reference to any specific large-language-model architecture or known model strengths (model-blind authoring). All items were reviewed by senior medical geneticists to confirm clarity, content validity, and difficulty calibration. Expert-authored questions were created *de novo* for this study, had not previously been published, and were not publicly released before model testing.

All questions were standardized to a 4-option format (A–D) with a single correct answer to eliminate structural bias across question sources. Both English- and Turkish-language versions were prepared: English-language questions were professionally translated to Turkish by a bilingual medical genetics specialist, with back-translation verification by an independent reviewer to ensure semantic equivalence. In response to peer review, the supplementary question files were re-audited against the complete item-level response records. All discrepancies proved to be clerical and did not affect the administered instrument: the numbering of the Turkish file was harmonized with the 50-item English sequence (a lost item number had displaced items 4–26 by one position), an inadvertently retained unnumbered draft item that was not among the administered questions was removed from both files, and option-set transcription defects were corrected. The exact English and Turkish item sets as administered and a definitive bilingual item-matching table with the answer keys are provided in the Supplementary Material.

### 2.3 Data collection and prompting

Each model was required to answer all 100 questions in English and Turkish. All questions were presented with the following zero-shot prompt without any step-by-step guidance or chain-of-thought instructions:

“*Respond to the following multiple-choice genetics questions by choosing the best answer for each item. For every numbered question, indicate only the letter (A, B, C, or D) corresponding to your selected option.”*

Each testing session was conducted as a fresh conversation session without access to previous responses. The English and Turkish versions were administered in separate sessions. All testing was performed between February 17 and 18, 2026 through each model's publicly available consumer chat interface using default settings (apart from the response-mode selections specified below), including default temperature and sampling parameters; web browsing, search, tool use, memory, file upload, and retrieval-augmented features were disabled where user-facing controls permitted, and no system or custom instructions were applied. The models were accessed through their developers’ publicly available consumer chat applications (ChatGPT, Gemini, Claude, Grok, and DeepSeek, respectively), and the model version designations displayed by each interface were recorded at the time of testing. Where an interface offered more than one user-selectable response mode or configuration under the same model name, the reasoning-enabled (“thinking”) configuration was selected for all administrations, as follows: GPT-5.2 was run in the “Thinking” mode of ChatGPT (rather than “Auto” or “Instant”); Gemini 3 Pro was accessed through the “Pro” reasoning-model selection in the Gemini model picker (previously labeled “Thinking with 3 Pro”); Claude Sonnet 4.6 was run with the extended-thinking toggle enabled; Grok 4 was run in “Expert” mode (rather than “Auto” or “Fast”); and DeepSeek-V3.2 was run with the “DeepThink” toggle enabled. No refusals, multi-letter, or otherwise malformed outputs occurred; every administration yielded a single, unambiguous answer that was scored directly against the answer key. The outputs were systematically recorded in a Microsoft Excel spreadsheet and evaluated against established correct answers. All English and Turkish questions with answer keys are provided in the Supplementary Material.

### 2.4 Statistical analyses

All analyses were performed in Python (version 3.12) with SciPy (version 1.17) and statsmodels (version 0.14). Because each of the 100 items was answered by all five models in both languages, the 1,000 binary outcomes are non-independent and clustered within items; accuracy estimates are therefore reported with Wilson 95% confidence intervals, which are presented as descriptive summaries only and do not reflect the dependence structure of the data, and between-condition effects were estimated with models that explicitly account for item-level clustering rather than with chi-square tests on pooled responses. The two equivalence analyses (question provenance and language) constituted the prespecified primary analyses; the between-model GEE contrasts were secondary analyses, corrected for multiplicity across the four comparisons against the reference model using the Holm procedure; and the McNemar, Fleiss *κ*, and error-concordance analyses were exploratory. Two complementary multilevel models were fitted. First, a generalized estimating equation (GEE) logistic regression with an exchangeable working correlation clustered on item modeled correctness as a function of question source (published vs. expert-authored), language (English vs. Turkish), and model, yielding cluster-robust, population-averaged odds ratios. Second, a Bayesian mixed-effects logistic regression with a random intercept for item and weakly informative priors was fitted as a prespecified sensitivity and robustness analysis for these sparse clustered data; the priors additionally regularize estimates under the quasi-complete separation expected at near-ceiling accuracy. Both models included additive main effects only: model-by-language, model-by-source, and source-by-language interaction terms could not be estimated stably from only 23 errors, so the reported effects are averages across the remaining factors and the conclusions are restricted accordingly. Model was treated as a fixed effect, with dependence among responses accounted for through item-level clustering; residual within-model dependence across items was not separately modeled, and inferences about individual models should be read with this in mind. Because the principal hypotheses concerned the absence of differences by question provenance and by language, we used two one-sided tests (TOST) of statistical equivalence with an *a priori* margin of ±5 percentage points, chosen before data collection to represent a difference of fewer than one item per 20 questions, judged educationally negligible; we acknowledge that this margin is broad relative to the overall error rate ultimately observed (2.3%), and the equivalence findings should be interpreted with this in mind. For the provenance comparison, each item's accuracy across its ten model–language administrations was treated as a single observation, and the difference between the 50 expert-authored and the 50 published items, with its standard error, was estimated from the between-item variability of these item-level accuracies (a two-sample comparison at the item level). For the language comparison, each item contributed a paired Turkish-versus-English accuracy difference across the five models, and the standard error was estimated from the distribution of these 100 paired item-level differences. In both analyses the item was the unit of analysis, respecting the clustering of the ten responses within each item, and equivalence was declared when the resulting item-clustered 90% confidence interval fell entirely within the margin. The item-paired language comparison was additionally tested with the exact McNemar test, both overall and within each model. Inter-model agreement on the selected answer option was quantified with Fleiss *κ*. Finally, an item-level error analysis examined whether incorrect responses were concordant (the same incorrect answer letter selected across models) or idiosyncratic. Two-sided *P* < 0.05 denoted significance unless otherwise stated.

## 3 Results

### 3.1 Overall accuracy and between-model differences

Pooled accuracy was 97.7% (977/1,000; Wilson 95% CI, 96.6–98.5). Model-specific accuracy ranged from 95.0% for Grok 4 to 99.5% for Gemini 3 Pro, with broadly overlapping confidence intervals (Table 1, Figure 1). In the item-clustered generalized estimating equation model, only Gemini 3 Pro differed nominally from the lowest-performing model, Grok 4 [odds ratio (OR), 10.38; 95% CI, 1.55–69.68; *P* = 0.016], with GPT-5.2 at the threshold (*P* = 0.055) and no other contrast reaching significance (Table 2); after Holm correction for the four secondary model comparisons, no contrast remained significant (corrected *P* = 0.064 for Gemini 3 Pro). The Bayesian sensitivity analysis showed a broadly similar ordering but was not identical to the GEE: the 95% credible intervals excluded 1 for both Gemini 3 Pro (OR, 14.30; 95% CrI, 2.89–70.91) and GPT-5.2 (OR, 7.71; 95% CrI, 2.01–29.52) versus Grok 4 (Table 2). Because only 12 of the 100 item clusters contained any error (Section 3.3), these estimates are highly imprecise, as reflected in the exceptionally wide interval estimates; between-model differences were minimal and should not be interpreted as a stable ranking.

**Figure 1:**
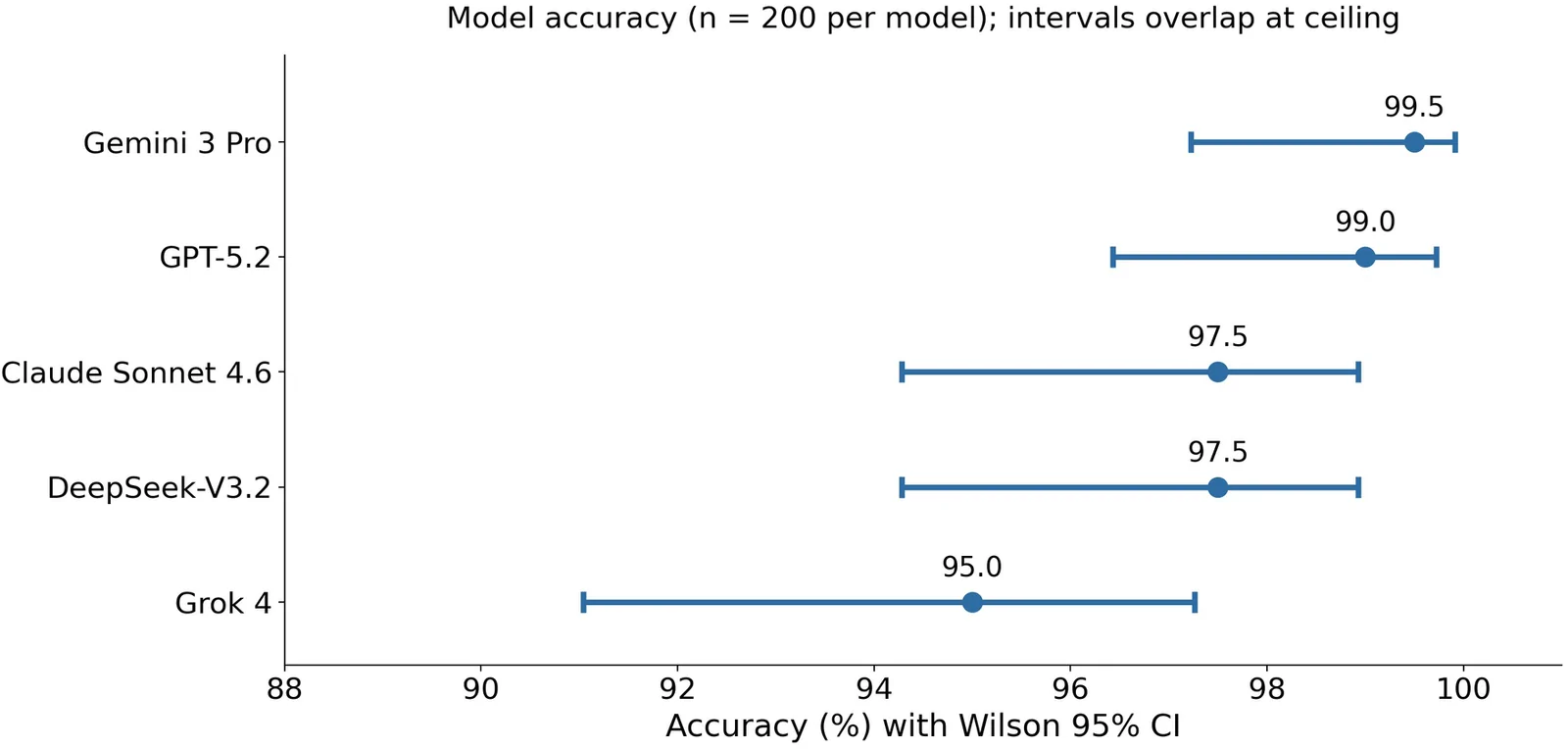
Accuracy of each model (*n* = 200) with wilson 95% confidence intervals. Intervals overlap substantially, reflecting near-ceiling performance.

**Table 1:** Model accuracy with wilson 95% confidence intervals (*n* = 200 per model).

Model	Correct	Accuracy %	95% CI (Wilson)
Gemini 3 Pro	199/200	99.5	97.2–99.9
GPT-5.2	198/200	99.0	96.4–99.7
Claude Sonnet 4.6	195/200	97.5	94.3–98.9
DeepSeek-V3.2	195/200	97.5	94.3–98.9
Grok 4	190/200	95.0	91.0–97.3

Each model answered 200 questions: 50 published and 50 expert-authored items, each administered in English and Turkish. Wilson intervals are descriptive only and do not account for item-level clustering (see Methods 2.4).

**Table 2:** Item-clustered regression of correctness: generalized estimating equation (cluster-robust) and Bayesian mixed-effects odds ratios (reference: grok 4).

Predictor	GEE OR (95% CI)	GEE P	Bayesian OR (95% CrI)
Source: expert-authored (ref published)	0.48 (0.11–2.07)	.329	0.60 (0.33–1.09)
Language: Turkish (ref English)	0.76 (0.42–1.37)	.366	0.85 (0.44–1.62)
DeepSeek-V3.2 (ref Grok 4)	2.05 (0.87–4.84)	.100	2.62 (0.98–7.01)
Claude Sonnet 4.6 (ref Grok 4)	2.05 (0.69–6.12)	.196	2.62 (0.98–7.01)
GPT-5.2 (ref Grok 4)	5.19 (0.97–27.94)	.055	7.71 (2.01–29.52)
Gemini 3 Pro (ref Grok 4)	10.38 (1.55–69.68)	.016	14.30 (2.89–70.91)

OR, odds ratio from a generalized estimating equation (exchangeable working correlation clustered on item); reference category, Grok 4. CI, confidence interval; CrI, credible interval. The Bayesian model was prespecified as a sensitivity analysis. Only 12 of 100 items contributed any error, so estimates are imprecise and should not be interpreted as a stable model ranking; the nominally significant Gemini 3 Pro contrast (*P* = .016) does not remain significant after Holm correction for the four model comparisons.

### 3.2 Question provenance and language: equivalence

In the item-clustered analysis, accuracy on expert-authored and published items was statistically equivalent within the pre-specified ±5-percentage-point margin (difference, −1.4 points; item-clustered 90% CI, −4.1 to +1.3; item-clustered two one-sided tests [TOST] *P* = 0.014). English and Turkish administrations were likewise equivalent (difference, −0.6 points; paired item-level TOST *P* < 0.001), and the item-paired McNemar test was non-significant overall (*P* = 0.58) and within every model (all *P* ≥ 0.99; model-specific discordant pairs are reported in the footnote to Table 3). Inter-model agreement on the exact option selected was high across all strata (Fleiss *κ*, 0.935–0.973), indicating convergence not only on accuracy but on identical answer choices. These analyses move the provenance and language comparisons beyond mere non-significance to positive evidence of equivalence at the pre-specified margin, with the caveat that the ±5-percentage-point margin is broad relative to the overall observed error rate of 2.3% and therefore constitutes a relatively permissive criterion.

**Table 3:** Equivalence testing (two one-sided tests, ±5 percentage-point margin) and paired mcNemar results.

Comparison	Difference	90% CI	*P* value	Equivalence (±5 pp)
Expert-authored vs. published	−1.4 pp	−4.1 to +1.3	.014	Established
Turkish vs. English (paired)	−0.6 pp	−1.8 to +0.6	<.001	Established
McNemar EN vs. TR (overall)	8 vs. 5 discordant	—	.58	n/a

pp, percentage points; TOST, two one-sided tests. *P* values in the first two rows are from the item-clustered TOST procedure described in Methods 2.4; the *P* value in the third row is from the exact McNemar test. Equivalence was declared when the item-clustered 90% CI fell entirely within ±5 percentage points. Discordant pairs are reported as EN-correct/TR-incorrect vs. EN-incorrect/TR-correct. Model-specific pairs (exact McNemar, all *P* ≥ 0.99): GPT-5.2 0 vs. 0; Gemini 3 Pro 1 vs. 0; Claude Sonnet 4.6 1 vs. 0; Grok 4 3 vs. 3; DeepSeek-V3.2 3 vs. 2.

### 3.3 Ceiling effect and item difficulty

Eighty-eight of 100 items were answered correctly in all ten administrations (item accuracy = 1.0), and 95 had item accuracy ≥ 0.9 (Figure 2); only five items had item accuracy < 0.9, including two at item accuracy = 0.50 (expert-authored item 34, a Bayesian carrier-risk calculation, and published item 6, the smallest human chromosome). The limited variance available for discrimination contextualizes the wide confidence intervals above and indicates that the multiple-choice format is approaching its measurement ceiling for current-generation models.

**Figure 2:**
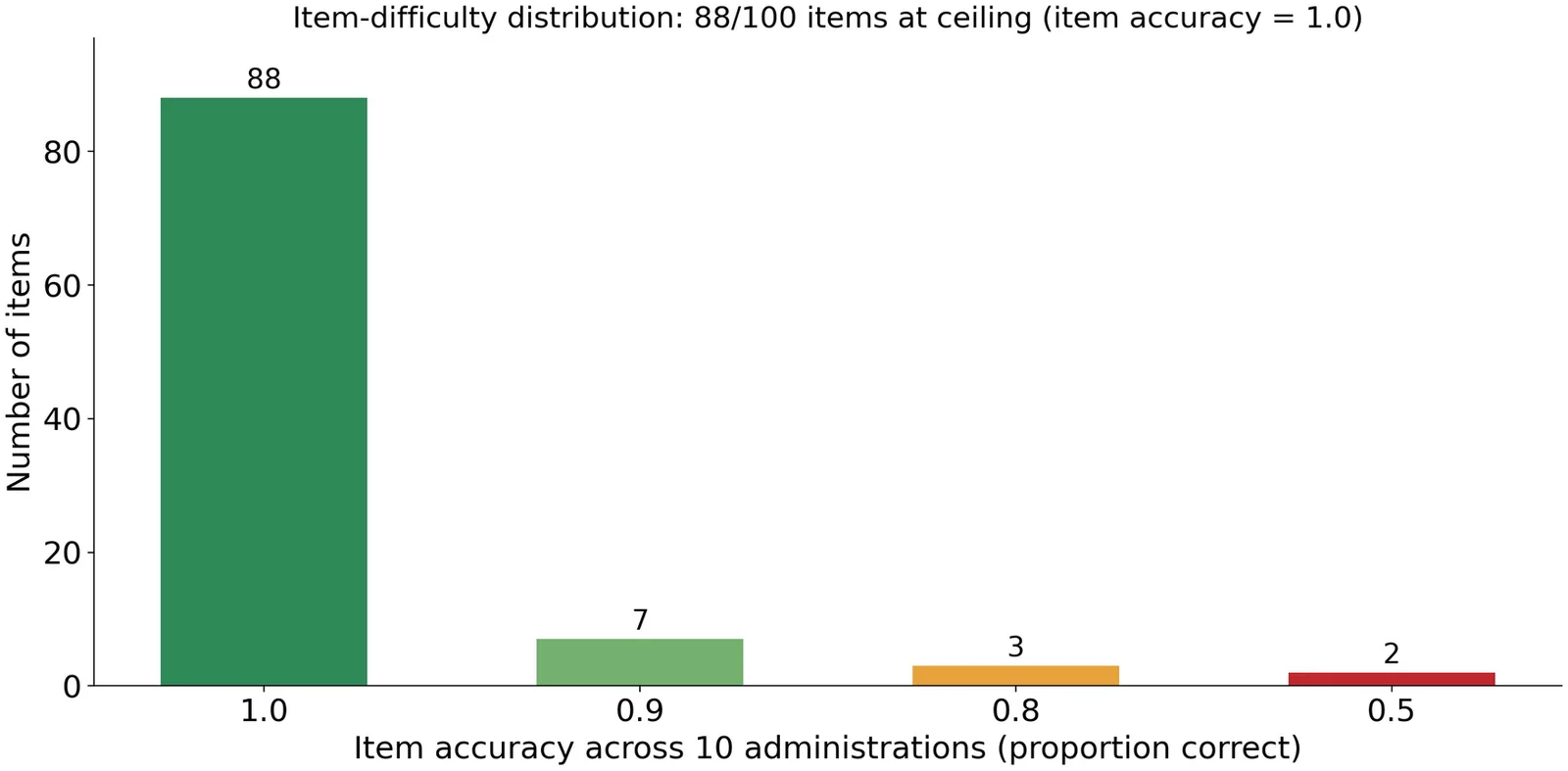
Item-difficulty distribution. Eighty-eight of 100 items were answered correctly in all ten administrations (item accuracy = 1.0); only five items fell below 0.9.

### 3.4 Concordant error patterns

Of the 1,000 responses, 23 (2.3%) were errors, concentrated in a small number of items (Table 4, Figure 3). All five items that elicited two or more errors failed concordantly, with every erring model selecting the same incorrect answer letter: published item 6 (all selecting chromosome 22 over the correct chromosome 21), expert-authored item 34 (all selecting the same incorrect Bayesian posterior), and expert-authored items 1, 11, and 33. Rather than dispersing across distractors, the distinct model families converged on a single incorrect answer in every multi-error item—a shared-error signature consistent with shared learned associations and/or overlapping training data, and suggestive of non-independent error patterns; the analysis cannot identify the underlying mechanism. This analysis was exploratory and *post hoc*: it rests on a small, retrospectively selected subset of five items, without a prespecified endpoint or a formal comparison against the concordance expected under independent errors, and should therefore be regarded as hypothesis-generating.

**Figure 3:**
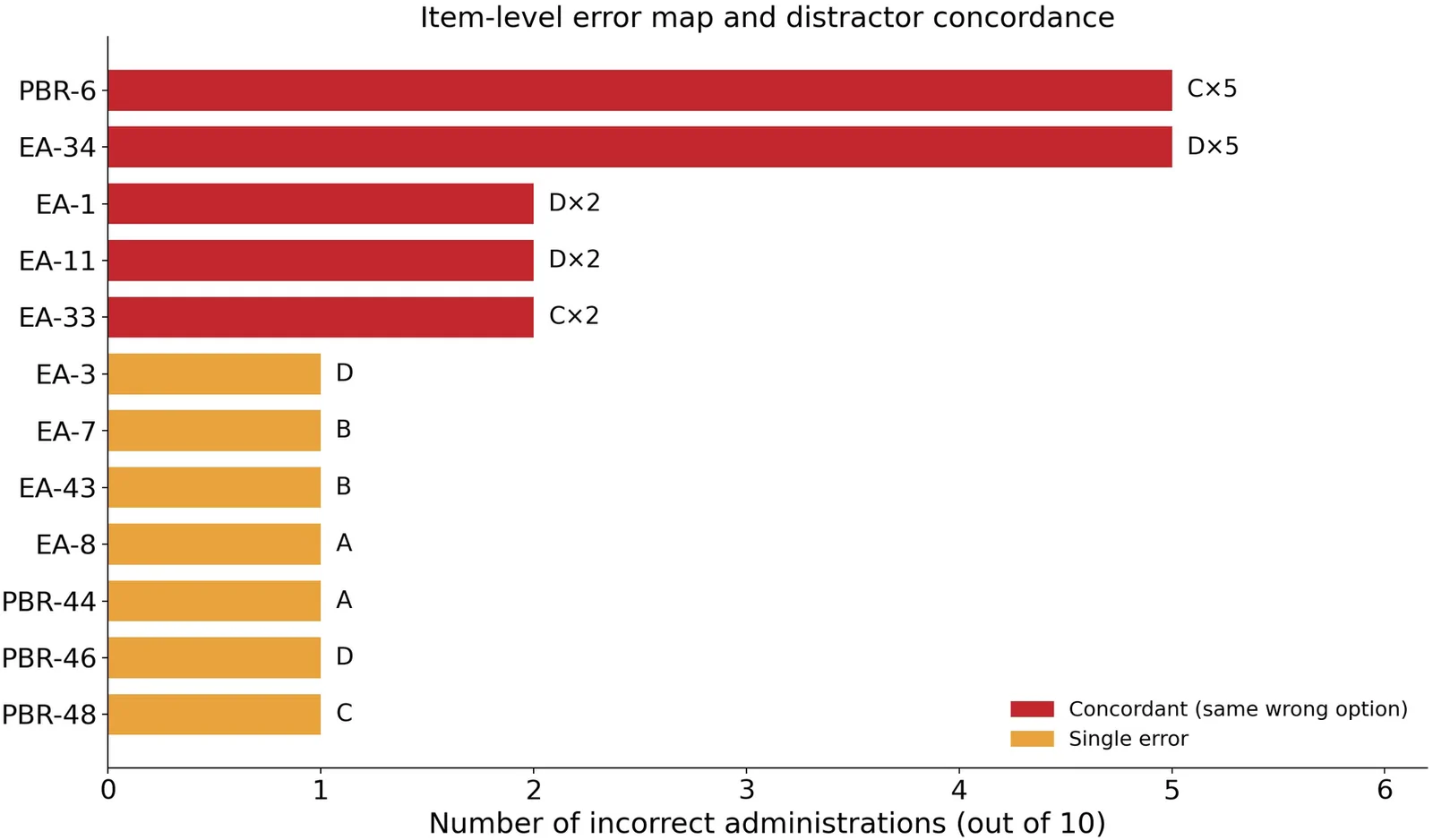
Item-level error map for the 12 items eliciting at least one error, ordered by error count. Every item with two or more errors failed concordantly, all erring models choosing the same incorrect option (shown beside each bar).

**Table 4:** Item-level error distribution and distractor concordance.

Item	Correct	Errors (n/10)	Pattern	Selected distractor(s)
PBR-6	B	5	Concordant	C,C,C,C,C
EA-34	C	5	Concordant	D,D,D,D,D
EA-1	B	2	Concordant	D,D
EA-11	C	2	Concordant	D,D
EA-33	D	2	Concordant	C,C
EA-3	C	1	Single	D
EA-7	C	1	Single	B
EA-43	D	1	Single	B
EA-8	B	1	Single	A
PBR-44	B	1	Single	A
PBR-46	B	1	Single	D
PBR-48	B	1	Single	C

EA, expert-authored; PBR, published board review. Each item was administered to 5 models   ×   2 languages = 10 combinations.

The two most error-prone items were tied at five errors each. The first was expert-authored Question 34 (Bayesian carrier risk calculation for Duchenne muscular dystrophy), which was answered incorrectly by 5 of the 10 model–language combinations. This item required sequential Bayesian updating: integrating a prior carrier probability of 1/2 (based on obligate carrier maternal status) with conditional evidence from three unaffected sons. The second, with the same error count, was published reference question 6 (“What is the smallest human chromosome?”), also failed by 5/10 combinations; all errors involved selecting chromosome 22 over the correct answer (chromosome 21), reflecting a well-known discrepancy between cytogenetic nomenclature and current sequence data. Together, these two items accounted for 10 of the 23 total errors (43.5%).

## 4 Discussion

This study provides one of the first systematic, head-to-head evaluations of multiple contemporary large-language models on medical genetics questions in a bilingual framework specifically designed to test the data contamination hypothesis. The findings are organized into five themes: near-ceiling accuracy with performance convergence; statistical equivalence across question provenance and language; convergence of distinct model families on identical answers; interpretable, concordant error patterns; and implications for clinical deployment.

### 4.1 Principal findings

All five models achieved 95.0–99.5% accuracy, with a mean of 97.7%. This represents a substantial improvement over earlier evaluations: Duong and Solomon reported 68.2% for ChatGPT (GPT-3.5) on comparable genetic content (10), and earlier-generation models likewise performed markedly lower on the Massive Multitask Language Understanding (MMLU) Medical Genetics benchmark (20). The improvement across model generations underscores the rapid maturation of large language model capabilities in structured biomedical knowledge domains. An intermediate step in this trajectory was documented by Flaharty et al., who evaluated both open- and closed-source models (including Claude, ChatGPT-3.5, and ChatGPT-4) on genetics condition identification tasks and found that larger, closed-source models substantially outperformed open-source alternatives. These results extend this finding to near-ceiling performance levels, suggesting that the performance gap between model families observed by Flaharty et al. may now be narrowing as contemporary models converge toward saturation (11).

### 4.2 Comparison with prior work

Notably, the genetics-specific accuracy observed here (97.7%) exceeds the performance levels reported in analogous benchmarking studies conducted in other medical specialties. For instance, a recent bilingual evaluation of nine large language models in psychiatry training reported a mean accuracy of 90.5% across the United States Medical Licensing Examination, Turkish Specialty Examination, and expert-authored items (21). This disparity likely reflects the more codified, rule-governed nature of genetics knowledge compared to disciplines such as psychiatry, where contextual reasoning, empathy, and theory-of-mind considerations introduce additional layers of complexity (22). In the item-clustered analysis, only the single best- versus worst-performing model contrast showed a nominal difference before multiplicity correction—a pattern of performance convergence that has been observed across medical specialties (21)—indicating that contemporary model families are reaching comparable proficiency at near-ceiling levels. Together, these observations suggest that current-generation large language models may be approaching the upper bounds of performance achievable through multiple-choice evaluation formats, a ceiling effect inherent to the testing paradigm rather than evidence of saturation in underlying reasoning capacity.

The absence of a significant accuracy difference between published reference items (98.4%) and novel, unpublished expert-authored items (97.0%; item-clustered GEE *P* = 0.33; Table 2) was confirmed as statistical equivalence within a pre-specified ±5-percentage-point margin (two one-sided tests), converting this comparison from a non-significant difference into positive, if margin-dependent, evidence against strong data contamination. Critics have argued that near-perfect large-language-model performance on established question banks may reflect the memorization of training data rather than genuine clinical reasoning (14, 15). This concern is not hypothetical: Flaharty et al. constructed their evaluation set from textbook-derived clinician descriptions, a design choice that, like the established board review question bank used in this study, raises plausible contamination concerns (11). Importantly, the finding that model accuracy on novel, unpublished items in our study was equivalent to published items (97.0% vs. 98.4%) provides indirect and limited evidence that the proficiency observed in both our study and that of Flaharty et al. may reflect generalized knowledge representation. This design mitigates this concern by incorporating 50 items that were absent from any published database at the time of testing. The finding that GPT-5.2 achieved 100% accuracy on the novel questions, equal to or exceeding its published-item accuracy, and that Grok 4 maintained 94% accuracy on these unpublished scenarios is consistent with a substantial component of generalized knowledge representation; it does not, however, exclude a contribution from memorization, because the novel items necessarily probe the same canonical genetics knowledge, may be structurally similar to existing educational materials, and the near-ceiling accuracy limits the sensitivity of the comparison. However, we note that this interpretation relies on an indirect proxy; a definitive resolution of the contamination question would require access to model training corpora, which is unavailable for proprietary systems.

Analogously, Flaharty et al. demonstrated that the same models maintained comparable accuracy across clinician-phrased and layperson-phrased formulations of the same genetics questions (11), a form of register-level invariance that parallels the language-level invariance observed here. Together, these findings suggest that contemporary models encode medical genetics knowledge in a form that is robust to both linguistic and register-level variation in query formulation. The absence of a significant language effect (English 98.0% vs. Turkish 97.4%; item-clustered GEE *P* = 0.37; McNemar *P* = 0.58), with all model-specific McNemar comparisons non-significant, suggests that contemporary large language models show cross-linguistic stability for this structured question-answering task. Equivalence was established within the prespecified ±5-percentage-point margin; however, near-ceiling accuracy and the relatively permissive margin limit inference regarding substantially narrower equivalence bounds. This finding aligns with Chimirri et al., who demonstrated consistent large language model performance in rare disease diagnosis across ten languages, including Turkish (19), and with the emerging consensus that question complexity rather than language is the primary determinant of performance variability (18, 21, 23). The practical implication is encouraging: genetics trainees in Turkish-medium programs can access large language model performance comparable to that of their English-medium counterparts for structured question-answering tasks.

### 4.3 Error patterns and cognitive boundaries

With only 23 errors across 1,000 responses, the qualitative analysis of error patterns provides arguably the most informative findings of this study. Errors were not randomly distributed but were concentrated in items with two distinct cognitive characteristics.

First, *multi-step probabilistic reasoning* proved challenging. Expert-authored Question 34 (Bayesian carrier risk for Duchenne muscular dystrophy) required the iterative application of Bayes’ theorem—integrating prior probability with conditional evidence across three offspring—a cognitive demand that has been consistently identified as a weakness in large language model evaluations across medical domains. Ogino and Wilson established Bayesian analysis as a cornerstone of genetic counseling (9); however, this quantitative reasoning demand exposes a fundamental limitation: large language models are trained on textual patterns and may not robustly execute the sequential numerical operations required for posterior probability calculations. This vulnerability is not unique to genetics; multi-step reasoning items have been shown to yield significantly lower accuracy than factual recall items across multiple medical disciplines (21). The genetics-specific finding reinforces this cross-domain vulnerability and highlights a boundary between pattern-based retrieval and genuine quantitative reasoning.

Second, *factual ambiguity arising from evolving scientific knowledge* generates systematic errors. Published reference question 6 exposed a tension between traditional cytogenetic teaching (chromosome 22 as the smallest) and current genomic data (chromosome 21 having fewer base pairs). Five of the ten model–language combinations defaulted to the historically prevalent—but outdated—answer, illustrating a specific large language model vulnerability: when established textbook knowledge conflicts with more recent data, models may default to the more frequently represented answer in their training corpus. This finding has implications for genetics education, in which rapidly evolving variant classifications and updated phenotype–genotype correlations may similarly outpace training data (24).

The near-ceiling accuracy across all five models supports the use of large language models as educational adjuncts in genetics training for formative assessment, concept review, question generation, and self-directed study. The comparable performance of all models—including those available for free—on structured factual tasks suggests that subscription-based systems may not be essential for foundational genetics education, a finding with implications for resource-limited settings. This finding contrasts with the earlier observation by Flaharty et al., who reported substantially lower performance for open-source models (Llama, Vicuna) compared to closed-source counterparts on genetics tasks (11), suggesting that the open/closed-source performance gap documented in 2024 has narrowed considerably within a single model generation, a trend with meaningful implications for equitable access to AI-assisted genetics education in resource-limited settings. However, the specific error patterns mandate caution: large language models should not be relied upon for clinical risk calculation tasks involving Bayesian reasoning or for questions where rapidly evolving knowledge may conflict with training data. In clinical genetic counseling, where precise recurrence risk estimation defines the core of professional practice (9), human oversight remains essential.

### 4.4 Convergence and concordant errors

A distinctive observation is that distinct model families converged not only on identical correct answers (Fleiss *κ* up to 0.97) but on identical incorrect answers on the hardest items. Every item that produced two or more errors failed concordantly: all erring models defaulted to chromosome 22 on the smallest-chromosome item and to the same incorrect posterior on the Bayesian carrier-risk item. This shared-error signature is consistent with shared learned associations across models while sharpening the boundary of that interpretation—where that representation encodes an outdated fact or an intuitive but incorrect probabilistic shortcut, all models inherit the same error. The clinical corollary is important: because the residual errors observed here were concordant rather than dispersed across distractors, using a second model as an independent check may offer limited protection on items of this type, and agreement across models cannot be treated as a proxy for correctness. This inference is exploratory: it rests on five retrospectively identified items and does not establish generally that consulting a second model provides little protection against error. The contribution is therefore not merely that these models are accurate, but that they are accurate and, when wrong, tend to be wrong in the same way.

### 4.5 Limitations

Several limitations warrant acknowledgment. First, the single-administration design precludes the assessment of test–retest reliability; prior work has demonstrated that large language model response consistency varies substantially across models, with intraclass correlation coefficients ranging from 0.65 to 0.93 (21). Second, binary scoring does not capture explanation quality, reasoning depth, or the clinical plausibility of incorrect responses. Third, the 100-item pool, while covering major genetics domains, may not fully represent the breadth of board examinations. Fourth, training data overlap between published reference questions and model corpora cannot be definitively excluded, although the null result on the published versus novel comparison provides indirect reassurance. Fifth, although we established statistical equivalence using two one-sided tests, this conclusion depends on the pre-specified ±5-percentage-point margin, which—though chosen *a priori* to represent an educationally negligible difference—is reviewer-contestable; a narrower margin or a larger, more difficult item pool would provide a more stringent test. Equivalence on accuracy also does not, by itself, exclude data contamination, because the novel items probe the same canonical knowledge in reworded form. Sixth, this study assessed text-based questions only; genetics practice increasingly involves the interpretation of visual data (pedigrees, cytogenetic images, and genomic plots) and variant classification using structured databases (24, 25), domains not assessed here. Seventh, the reported models include additive main effects only; because interaction terms could not be estimated from the sparse errors, the equivalence of provenance and language effects has not been demonstrated separately within each individual model. Eighth, the between-model contrasts rest on only 12 items that elicited any error and are correspondingly unstable, and the Wilson intervals in Table 1 are descriptive only; accordingly, near-perfect accuracy on this four-option multiple-choice benchmark should not be equated with readiness for clinical genetic counseling, variant interpretation, or quantitative risk assessment.

Future research should transition from static multiple-choice benchmarks toward dynamic reasoning evaluation: variant interpretation tasks with explicit stepwise reasoning, pedigree analysis requiring quantitative risk assessment, and integration of genomic database information through retrieval-augmented generation approaches (25). Longitudinal tracking of large language model performance across model generations would provide insights into the trajectory of artificial intelligence capabilities in this rapidly advancing field.

## 5 Conclusion

This study demonstrates that contemporary large language models achieve near-ceiling accuracy on structured medical genetics questions, with no detectable difference by question provenance or administration language. Accuracy was statistically equivalent across question provenance and language, and distinct model families converged not only on identical correct answers but on identical errors on the hardest items, a shared-error signature consistent with shared learned associations and/or overlapping training data, although this indirect evidence cannot identify the underlying mechanism. However, the concentration of errors in items requiring Bayesian probabilistic reasoning and involving evolving scientific knowledge reveals interpretable cognitive boundaries. Performance on this restricted multiple-choice benchmark should not be equated with clinical reliability. These findings support a purpose-driven deployment model in which large language models serve as educational adjuncts in genetics training, whereas human oversight remains essential for complex risk assessment and clinical genetic counseling.

## Data Availability

The raw data supporting the conclusions of this article will be made available by the authors, without undue reservation.
