# Supplementary material for: Benchmarking five large language models in medical genetics: a bilingual comparative evaluation using published and novel expert-authored questions
Source: Front Med (Lausanne). 2026 Sep 15;13:1938886. doi: 10.3389/fmed.2026.1938886 (PMC13619396; doi:10.3389/fmed.2026.1938886)
Supplement: Supplementary file 1 [file Datasheet1.docx]

**Supplementary Material**

**Benchmarking Five Large Language Models in Medical Genetics:**

**A Bilingual Comparative Evaluation Using Published and Novel Expert-Authored Questions**

**Question Sets:**

PBR = Published Board Review questions (source: Solomon BD. Medical Genetics and Genomics: Questions for Board Review. Wiley; 2022 - topics listed in Section 1; full text not reproduced)

EA = Expert-Authored novel questions (Created de novo for this study)

**Section 1: Published Board Review (PBR) Items - Source and Topics**

The 50 published board-review items were drawn from: Solomon BD. Medical Genetics and Genomics: Questions for Board Review. Hoboken, NJ: John Wiley & Sons; 2022. To respect the copyright of the source, the full text of these items is not reproduced here; the questions are available in the source textbook. The topic assessed by each item is listed below; item numbers (PBR 1–50) correspond to the identifiers used in the analysis. The 50 novel, expert-authored items are reproduced in full in the following sections.

1. Number of protein-coding genes in the human genome

2. Proportion of the genome represented by the exome

3. Germ layer of origin of the central nervous system

4. Terminology for sequence-independent (epigenetic) DNA modification

5. Identifying a molecule that is not a DNA nucleotide base

6. Smallest human chromosome (cytogenetic teaching vs genomic base-pair size)

7. Terminology for transcription of DNA into RNA

8. Terminology for programmed cell death

9. Diagnosis suggested by neonatal puffy hands and feet

10. Cranial nerves involved in Moebius sequence

11. Metabolic condition mimicking Marfan-like features

12. Characteristic physical sign of Wilson disease

13. Trisomy associated with polydactyly and midline cleft lip

14. Body-odour description in primary trimethylaminuria

15. Terminology distinguishing telecanthus from hypertelorism

16. Terminology for repetitive involuntary eye movements

17. Earliest typical clinical feature of neurofibromatosis type 1 in infancy

18. Condition with neonatal hypotonia followed by childhood hyperphagia

19. Condition with thrombocytopenia, eczema and recurrent infections

20. Electrolyte abnormality typical of 22q11.2 deletion syndrome

21. Condition indicated by the brain MRI molar-tooth sign

22. Condition most associated with supravalvar aortic stenosis

23. Most common eye finding in CHARGE syndrome

24. Tumour-predisposition syndrome with haemangioblastoma, renal cell carcinoma and phaeochromocytoma

25. Condition presenting with haematuria and hearing loss

26. Condition caused by absent enteric ganglion cells in the colon

27. SALL1-related syndrome with anorectal, ear and thumb anomalies

28. Condition associated with the prenatal ultrasound lemon sign

29. Gene associated with hereditary diffuse gastric cancer

30. Gene most commonly implicated in hereditary prostate cancer

31. Most likely causative gene in Noonan syndrome

32. Syndrome from 5q35 microdeletion or NSD1 variant

33. Condition associated with low TREC on newborn screening

34. Allele underlying sickle cell disease

35. X-linked condition caused by ABCD1 variants

36. Enzyme deficiency underlying Gaucher disease

37. Gene affected by repeat expansion in myotonic dystrophy type 1

38. Gene underlying Hutchinson-Gilford progeria syndrome

39. Karyotype corresponding to Klinefelter syndrome

40. X-linked condition from severe HGPRT deficiency

41. Condition caused by galactocerebrosidase deficiency

42. Most beneficial treatment in familial Mediterranean fever

43. Condition with an approved gene therapy

44. Most common trigger of haemolysis in G6PD deficiency

45. Treatment for ALDH7A1-related (pyridoxine-dependent) epilepsy

46. Recurrence risk for the child of an X-linked recessive carrier

47. Identifying a condition with X-linked inheritance

48. Identifying a repeat disorder with autosomal recessive inheritance

49. Terminology for uniparental disomy

50. Terminology for genetic anticipation

# Section 2: Expert-Authored (EA) Questions - English

These questions were created de novo by clinical genetics experts for this study and were absent from any published database at the time of testing. Each item is 4-option single-best-answer format. Correct answers are indicated.

1. A 3-year-old male is referred to the genetics clinic due to severe failure to thrive and global developmental delay. His medical history is significant for polyhydramnios and premature birth. Since infancy, he has required gastrostomy tube feeding due to severe oral feeding difficulties.

Physical examination reveals short stature, coarse facial features with full lips and a large mouth, and sparse, curly hair. The skin is soft and loose, with notably deep creases on the palms and soles. Musculoskeletal evaluation shows ulnar deviation of the wrists and fingers. A recent cardiac evaluation identified hypertrophic cardiomyopathy, and the electrocardiogram captured a chaotic atrial rhythm (multifocal atrial tachycardia).

**Which of the following is the most frequent malignant tumor associated with this syndrome?**

A) Neuroblastoma

B) Rhabdomyosarcoma

C) Hepatoblastoma

D) Wilms tumor

Correct Answer: B

2. A couple presents to the genetics clinic due to a history of recurrent spontaneous abortions. Physical examination of both partners is unremarkable. Cytogenetic analysis reveals a normal 46,XX karyotype in the female partner. The male partner’s karyotype is reported as 45,XY,der(13;14)(q10;q10). The couple is concerned about the health of their future children.

**Which of the following is the approximate empiric risk of having a live-born child with an unbalanced chromosomal abnormality for this couple?**

A) < 1%

B) 10-15%

C) 33%

D) 50%

Correct Answer: A

3. A couple presents to the genetics clinic for counseling regarding future reproductive options. They have a 5-year-old son diagnosed with Fanconi anemia, confirmed to be due to compound heterozygous pathogenic variants in the FANCA gene. The parents wish to conceive another child who could serve as a hematopoietic stem cell donor for their affected son. They inquire about the likelihood of a future sibling being a suitable donor.

**Assuming the FANCA gene and the HLA complex segregate independently, what is the probability that their next child will be phenotypically unaffected by Fanconi anemia and also be an HLA-identical match to the affected sibling?**

A) 3/4 (75%)

B) 1/4 (25%)

C) 3/16 (18.75%)

D) 1/8 (12.5%)

Correct Answer: C

4. A 29-year-old female presents to the genetics clinic for preconception counseling. She and her husband are planning to start a family soon but have concerns regarding her family history of fertility issues. She reports that her mother experienced menopause at age 38, and her maternal aunt experienced menopause at age 36. The patient has regular menstrual cycles, but recent hormonal testing revealed a borderline elevated follicle-stimulating hormone (FSH) level.

**Given the family history of premature ovarian insufficiency (POI), which of the following molecular tests is most indicated to assess the risk of intellectual disability in her future male offspring?**

A) Thrombophilia panel (e.g., Factor V Leiden, Prothrombin)

B) *FMR1* CGG triplet repeat analysis

C) Peripheral blood karyotype

D) Chromosomal Microarray Analysis (CMA)

Correct Answer: B

5. A 22-year-old female is referred to the genetics clinic for the management of Marfan syndrome, confirmed by a pathogenic heterozygous variant in the *FBN1* gene. Her medical history includes high myopia and ectopia lentis. Physical examination reveals tall stature, arachnodactyly, and a pectus excavatum deformity. A recent echocardiogram shows an aortic root Z-score of +3.5, indicating significant dilation.

To slow the rate of aortic root dilation and reduce hemodynamic stress, the cardiologist initiates pharmacologic therapy. Which of the following classes of medication is considered the standard first-line treatment for this purpose?

A) Calcium channel blockers

B) Beta-adrenergic receptor blockers

C) HMG-CoA reductase inhibitors (Statins)

D) Loop diuretics

Correct Answer: B) Beta-adrenergic receptor blockers

6. A 42-year-old male presents with recurrent nephrolithiasis. Laboratory evaluation reveals an albumin-corrected calcium level of 11.2 mg/dL and an elevated parathyroid hormone (PTH) level. His medical history is significant for a 20 pack-year smoking history. Family history is notable for his father passing away from a pancreatic tumor at a young age. Genetic testing confirms a pathogenic variant in the *MEN1* gene. The patient is scheduled for a subtotal parathyroidectomy for the management of primary hyperparathyroidism.

**Given the patient's diagnosis and specific risk factors, which of the following prophylactic procedures should be discussed and performed during the surgery to reduce the risk of an aggressive malignancy?**

A) Prophylactic transcervical thymectomy

B) Prophylactic distal pancreatectomy

C) Prophylactic splenectomy

D) Prophylactic bilateral adrenalectomy

Correct Answer: A) Prophylactic transcervical thymectomy

7. A 45-year-old male is referred to the nephrology clinic due to incidental findings on an abdominal ultrasound. His medical history is significant for two episodes of spontaneous pneumothorax in his 30s. On physical examination, multiple small, skin-colored, dome-shaped papules are noted on his face, particularly around the nose and cheeks, as well as on his neck. A subsequent abdominal MRI reveals bilateral, multifocal solid renal masses ranging from 2 to 3 cm in diameter.

**Given the patient’s clinical presentation and the most likely underlying genetic syndrome, which of the following histological subtypes is the most characteristic finding for these renal tumors?**

A) Clear cell renal cell carcinoma

B) Angiomyolipoma

C) Hybrid oncocytic tumor (chromophobe and oncocytoma features)

D) Type 1 papillary renal cell carcinoma

**Correct Answer: C) Hybrid oncocytic tumor (chromophobe and oncocytoma features)**

8. A 34-year-old female is referred to the genetics clinic due to a history of multiple trichilemmomas and a multinodular goiter requiring thyroidectomy. Her physical examination is notable for macrocephaly (head circumference >98th percentile) and oral papillomas. Genetic testing confirms a heterozygous pathogenic variant in the PTEN gene, consistent with Cowden syndrome. During the post-test counseling, the patient asks about her asymptomatic 7-year-old son, whose head circumference is also noted to be above the 98th percentile.

**Which of the following is the most appropriate recommendation regarding genetic testing for the 7-year-old son?**

A) Testing should be deferred until age 18, as Cowden syndrome is primarily an adult-onset cancer predisposition syndrome.

B) Testing is recommended now, as surveillance for thyroid cancer should begin at age 7.

C) Testing should be offered only if the child develops signs of autism spectrum disorder or developmental delay.

D) Testing is not indicated, as a clinical diagnosis can be made based solely on the presence of macrocephaly.

Correct Answer: B

9. A 5-year-old male is referred to the pediatric genetics clinic for follow-up of short stature. He was diagnosed with achondroplasia in infancy, confirmed by a heterozygous pathogenic variant (c.1138G>A) in the *FGFR3* gene. Physical examination reveals frontal bossing, midfacial retrusion, rhizomelic shortening of the limbs, and a trident hand configuration. The parents are interested in new pharmacological options.

**Which of the following agents is the first and currently only FDA-approved therapy specifically indicated to increase linear growth in pediatric patients with achondroplasia?**

A) Somatropin

B) Vosoritide

C) Pamidronate

D) Burosumab

**Correct Answer: B) Vosoritide**

10. A newborn male is evaluated in the neonatal intensive care unit due to macrosomia (birth weight >97th percentile) and hypoglycemia. Physical examination reveals macroglossia, a large omphalocele requiring surgical closure, and distinctive linear creases on the earlobes. Abdominal ultrasound identifies hepatomegaly and enlarged kidneys.

**Which of the following is the most appropriate *initial* genetic test to confirm the diagnosis in this patient?**

A) Chromosomal Microarray Analysis (CMA)

B) Methylation analysis of the 11p15.5 region (e.g., MS-MLPA)

C) Karyotype analysis

D) Whole Exome Sequencing (WES)

**Correct Answer: B**

11. A 34-year-old male is referred to the medical genetics clinic for counseling after his mother passed away from pancreatic carcinoma. His family history is significant for a maternal aunt with breast cancer diagnosed at age 45 and a maternal uncle with prostate cancer. Genetic testing confirms a pathogenic germline variant in the *BRCA2* gene. During the counseling session, the patient expresses concern about his personal risk of developing breast cancer.

**Based on his specific genetic result (*BRCA2*), which of the following represents the approximate lifetime risk for breast cancer in this patient?**

A) < 0.1% (General population risk) B) 1% - 2% C) 6% - 8% D) 15% - 20%

**Correct Answer: C**

12. A 62-year-old male with Stage III colon cancer initiates adjuvant chemotherapy with the FOLFOX regimen (5-Fluorouracil, Leucovorin, and Oxaliplatin). Ten days after his first infusion, he presents to the emergency department in critical condition. Physical examination reveals severe stomatitis and diffuse abdominal tenderness. Laboratory results show Grade 4 neutropenia (ANC < 500/µL), thrombocytopenia, and acute kidney injury due to severe dehydration from watery diarrhea. The oncologist suspects a pharmacogenetic defect affecting drug metabolism.

**Which of the following germline genetic variants is the most likely cause of this patient’s severe, life-threatening toxicity?**

A) *UGT1A1* *28 allele homozygosity

B) *DPYD* loss-of-function variant

C) *KRAS* exon 2 mutation

D) *TPMT* deficiency

**Correct Answer: B) *DPYD* loss-of-function variant**

13. **A 6-year-old male with a history of recurrent pulmonary infections and steatorrhea is diagnosed with Cystic Fibrosis following a positive sweat test. Molecular genetic analysis reveals the G551D (Class III, Gating) mutation. The pediatric pulmonologist decides to initiate Ivacaftor, a targeted therapy addressing the pathophysiology of this specific mutation.**

**Which of the following best describes the molecular mechanism of action of this drug?**

A) It hydrolyzes extracellular DNA in the airway, thereby reducing mucus viscosity.

B) It acts as a chaperone to prevent the degradation of misfolded CFTR protein, facilitating its trafficking to the cell surface (Corrector).

C) It increases the probability of the chloride channel opening for CFTR proteins that are already present at the cell surface (Potentiator).

D) It inhibits the epithelial sodium channel (ENaC) to increase airway surface liquid hydration.

Correct Answer: C) It increases the probability of the chloride channel opening for CFTR proteins that are already present at the cell surface (Potentiator).

14. A 2-year-old male is referred to the genetics clinic due to a history of seizures. The seizures began at 6 months of age and were initially triggered by fever. Over time, he developed generalized tonic-clonic and hemiclonic seizures that also occur without fever. His prenatal and birth history are unremarkable. Physical examination reveals no dysmorphic features or neurocutaneous stigmata. Cranial MRI is reported as normal. His neuromotor development was age-appropriate until the onset of seizures, after which a mild stagnation was observed. **In a panel test ordered with a preliminary diagnosis of "isolated genetic epilepsy," a pathogenic variant in which of the following genes is most likely to be identified?**

A) *SCN1A*

B) *TSC1*

C) *FMR1*

D) *KCNQ2*

Correct Answer: A

15. A 3-day-old neonate is admitted to the intensive care unit due to frequent focal tonic seizures. The infant’s neurological examination is normal during the interictal period. Family history reveals that the father had similar seizures during infancy which resolved spontaneously within a few months. Cranial MRI and metabolic screening are unremarkable. **Which of the following is the most likely genetic etiology for this clinical presentation (Benign Familial Neonatal Epilepsy)?**

A) *KCNQ2* B) *MECP2* C) *POLG* D) *CHRNA4*

Correct Answer: A

16. A 4-year-old female is evaluated for drug-resistant epilepsy and developmental delay. The seizures reportedly began at 1 year of age and were triggered by fever. There is no family history of epilepsy. Genetic analysis identifies a pathogenic variant in the ***PCDH19*** gene. **Which of the following statements regarding the inheritance pattern and clinical features of the epilepsy syndrome associated with this gene is correct?**

A) It is X-linked, but the disease primarily manifests in heterozygous females (Male Sparing). B) It is autosomal recessive and seen only in consanguineous families.

C) It shows mitochondrial inheritance and is transmitted only maternally.

D) It shows Y-linked inheritance and is seen only in males.

Correct Answer: A

17. A patient followed for isolated epilepsy develops resistance to treatment. A detailed skin examination using a Wood’s lamp reveals three hypopigmented macules (ash-leaf spots). A subsequent cranial MRI demonstrates cortical tubers.

**In this patient, a defect is expected in the inhibitor genes (TSC1 or TSC2) of which of the following signaling pathways?**

A) mTOR pathway

B) Wnt signaling pathway

C) Sonic Hedgehog pathway

D) GABAergic inhibition pathway

Correct Answer: A

18. A patient presents with absence-like staring spells, myoclonic seizures, and global developmental delay starting in infancy. It is observed that the seizures are specifically triggered by **fasting** and respond dramatically to a ketogenic diet. CSF analysis reveals a low glucose level (hypoglycorrhachia) despite normal blood glucose. **What is the most likely genetic diagnosis?**

A) *SLC2A1* (GLUT1 Deficiency Syndrome)

B) *CACNA1H*

C) *SCN2A*

D) *ALDH7A1*

Correct Answer: A

19. A 38-year-old male is diagnosed with colorectal cancer. His family history is significant for colon and endometrial cancers in multiple generations. Germline genetic testing confirms a pathogenic heterozygous variant in the *MSH2* gene, consistent with a diagnosis of Lynch Syndrome. During the genetic counseling session, he requests that his asymptomatic 10-year-old daughter be tested for the familial mutation "so we can know what to expect.

**According to professional ethical guidelines (ACMG/NSGC) regarding genetic testing of minors for adult-onset conditions, which of the following is the most appropriate recommendation?**

A) Testing should be deferred until the daughter reaches the age of majority (typically 18 years), as surveillance interventions usually begin in early adulthood.

B) Testing should be performed immediately, as screening colonoscopies for Lynch Syndrome are recommended to begin at age 10.

C) Testing is recommended now to alleviate parental anxiety, even if no medical intervention is indicated during childhood.

D) Testing should be performed now because Lynch Syndrome also carries a high risk for childhood leukemia and brain tumors.

Correct Answer: A) Testing should be deferred until the daughter reaches the age of majority (typically 18 years), as surveillance interventions usually begin in early adulthood.

20. A 17-year-old female is referred to the endocrinology clinic after routine blood work revealed an elevated fasting glucose of 135 mg/dL and an HbA1c of 7.2%. She is non-obese and has no autoantibodies associated with Type 1 diabetes. Her medical history is notable for recurrent urinary tract infections. Family history reveals that her father was diagnosed with diabetes at age 25 and developed end-stage renal disease requiring dialysis in his 30s.

An abdominal ultrasound is performed, showing a hypoplastic pancreas with atrophy of the body and tail. Additionally, bilateral renal cortical cysts and a hypoplastic left kidney are identified. Laboratory review also notes persistent, unexplained hypomagnesemia.

Given the clinical presentation and family history, which of the following genes is the most likely cause of this patient’s condition?

A) HNF1A (MODY 3) B) GCK (MODY 2) C) HNF1B (MODY 5) D) HNF4A (MODY 1)

Correct Answer: C

21. A male newborn is evaluated in the neonatal intensive care unit for significant proximal limb shortening (rhizomelia) and micrognathia. Ophthalmologic examination reveals bilateral congenital cataracts. Skeletal radiographs demonstrate extensive punctate calcifications (stippling) in the humeral and femoral epiphyses, along with coronal clefts of the vertebral bodies. Biochemical analysis reveals normal plasma Very Long Chain Fatty Acid (VLCFA) levels, but markedly reduced red blood cell plasmalogen levels and age-dependent elevation of phytanic acid.

**Based on the clinical and biochemical findings, which of the following is the most likely genetic defect and the associated cellular organelle?**

A) PEX1 gene – Peroxisome (Zellweger Spectrum Disorder)

B) EBP gene – Endoplasmic Reticulum (X-linked Dominant Chondrodysplasia Punctata)

C) PEX7 gene – Peroxisome (Rhizomelic Chondrodysplasia Punctata Type 1)

D) ARSE gene – Golgi Apparatus (X-linked Recessive Chondrodysplasia Punctata)

Correct Answer: C) *PEX7* gene – Peroxisome (Rhizomelic Chondrodysplasia Punctata Type 1)

22. A 39-year-old female undergoes Chorionic Villus Sampling (CVS) at 11 weeks gestation due to advanced maternal age. The cytogenetic result is reported as Trisomy 15 (47,XX,+15). To confirm the diagnosis, an amniocentesis is performed at 16 weeks, which reveals a normal female karyotype (46,XX). The pregnancy is continued to term. The newborn presents with severe hypotonia, poor feeding, and hypogonadism, highly suggestive of Prader-Willi syndrome.

**Based on the discrepancy between the prenatal tests and the subsequent clinical presentation, which of the following cellular mechanisms best explains the normalization of the fetal karyotype resulting in this specific outcome?**

A) Monosomy rescue B) Gamete complementation C) Trisomy rescue D) Post-fertilization mitotic nondisjunction

**Correct Answer: C) Trisomy rescue**

23. **A 17-year-old phenotypic female presents to the clinic with a chief complaint of primary amenorrhea. She has never had a menstrual period. Her past medical history is unremarkable.**

**Physical examination reveals:**

- **Height:** 50th percentile.
- **Breast Development:** Tanner Stage 4 (Normal/Well-developed).
- **Pubic/Axillary Hair:** Tanner Stage 1 (Sparse to absent).
- **Genital Exam:** Normal female external genitalia with a shortened, blind-ending vaginal pouch.

**Pelvic ultrasound fails to visualize a uterus or fallopian tubes but identifies bilateral solid masses within the inguinal canals. A subsequent chromosomal analysis reveals a 46,XY karyotype.**

**Which of the following is the most likely underlying etiology for this patient’s clinical presentation?**

A) Gonadal dysgenesis due to an *SRY* gene deletion (Swyer syndrome) B) Defect in the androgen receptor due to an *AR* gene mutation (Complete Androgen Insensitivity Syndrome) C) Defect in the conversion of testosterone to dihydrotestosterone due to *SRD5A2* deficiency D) Agenesis of the Müllerian ducts due to a developmental defect (Mayer-Rokitansky-Küster-Hauser syndrome)

**Correct Answer: B) Defect in the androgen receptor due to an *AR* gene mutation (Complete Androgen Insensitivity Syndrome)**

24. **A 26-year-old postpartum female presents to the cardiology clinic after experiencing a syncopal episode. She reports that the event occurred early in the morning when she was suddenly startled by her alarm clock ringing. She denies any chest pain or palpitations during exercise. Her family history is significant for a sister who died suddenly at age 30 during a period of emotional stress. An ECG reveals a corrected QT interval (QTc) of 510 ms with low-amplitude, notched T waves.**

**Based on the specific trigger (auditory stimulus) and the ECG findings, a loss-of-function pathogenic variant in which of the following genes is the most likely cause?**

A) *KCNQ1* (LQT1) B) *KCNH2* (LQT2) C) *SCN5A* (LQT3) D) *ANK2* (LQT4)

Correct Answer: B) *KCNH2* (LQT2)

25. **A 34-year-old male presents to the neurology clinic with complaints of progressive muscle stiffness and difficulty relaxing his grip after shaking hands. He also reports early-onset cataracts and significant fatigue. On physical examination, he has frontal balding, temporal wasting, and bilateral ptosis. When the thenar eminence is struck with a reflex hammer, a sustained muscle contraction is observed. Genetic testing confirms a CTG trinucleotide repeat expansion (>100 repeats) in the 3' untranslated region of the *DMPK* gene.**

**Which of the following molecular mechanisms best explains the pathogenesis of the myotonia observed in this patient?**

A) Loss of function mutations in the *CLCN1* gene leading to reduced chloride conductance. B) Toxic gain-of-function of the mutant RNA, causing aberrant splicing of the *CLCN1* pre-mRNA. C) Autoimmune destruction of the postsynaptic acetylcholine receptors at the neuromuscular junction. D) Gain-of-function mutation in the *SCN4A* gene causing delayed inactivation of sodium channels.

Correct Answer: B) Toxic gain-of-function of the mutant RNA, causing aberrant splicing of the *CLCN1* pre-mRNA.

26. A 25-year-old male presents to the neurology clinic with complaints of resting tremor in his hands, dysarthria, and increasing clumsiness. His medical history is notable for unexplained elevations in liver function tests. An ophthalmologic examination reveals Kayser-Fleischer rings in the cornea.

**Which of the following is the most likely diagnosis and the expected laboratory finding for this patient?**

A) Hemochromatosis – Increased transferrin saturation

B) Wilson Disease – Decreased serum ceruloplasmin level

C) Huntington Disease – Increased CAG repeat number

D) Alcoholic Liver Disease – Elevated GGT

**Correct Answer:** B (Wilson Disease – Decreased serum ceruloplasmin level)

27. A 2-month-old female infant is brought to the genetics clinic for evaluation of a congenital anomaly. The infant was born with a left-sided cleft lip and palate, which is currently being managed by a craniofacial team. On physical examination, two small, bilateral paramedian depressions (sinuses) are noted on the vermilion border of the lower lip. Family history reveals that the patient's father has similar depressions on his lower lip but no history of a cleft.

**Based on the clinical findings and family history, which of the following is the most likely diagnosis?**

A) Isolated (Non-syndromic) Cleft Lip and Palate

B) Van der Woude syndrome

C) 22q11.2 deletion syndrome (DiGeorge/Velocardiofacial)

D) Trisomy 13 (Patau syndrome)

**Correct Answer: B) Van der Woude syndrome**

28. A 12-year-old female is referred to the genetics clinic by her dentist due to the persistence of primary teeth and failure of permanent teeth to erupt. A panoramic dental radiograph reveals numerous supernumerary (impacted) teeth and cyst formation around the unerupted molars.

On physical examination, the patient has mild short stature. Craniofacial features include a prominent forehead (frontal bossing), hypertelorism, and a palpable soft spot indicating a persistent open anterior fontanelle. Musculoskeletal examination demonstrates narrow, sloping shoulders with excessive mobility, allowing the patient to approximate her shoulders anteriorly to the midline**.**

**Based on these characteristic clinical findings, which of the following is the most likely diagnosis?**

A) Osteogenesis Imperfecta

B) Pycnodysostosis

C) Cleidocranial dysplasia

D) Achondroplasia

Correct Answer: C) Cleidocranial dysplasia

29. **A 14-year-old female is referred to the endocrinology clinic due to short stature and delayed puberty. Physical examination reveals a round face, obesity, and subcutaneous ossifications. A hand X-ray demonstrates distinct shortening of the 4th metacarpals (brachydactyly type E).**

**Laboratory evaluation reveals the following:**

- **Calcium:** Low
- **Phosphorus:** High
- **Parathyroid Hormone (PTH):** Elevated
- **TSH:** Elevated (with normal free T4)
- **FSH:** Elevated

**Based on the clinical phenotype (Albright Hereditary Osteodystrophy) and the laboratory evidence of multi-hormone resistance, a loss-of-function mutation in which of the following imprinted genes is the most likely cause?**

A) *FGFR3* B) *SHOX* C) *GNAS* D) *AIRE*

Correct Answer: C) GNAS

30. A 48-year-old male presents with fatigue, arthralgia, and loss of libido. He has a recent diagnosis of diabetes mellitus. Physical examination reveals generalized hyperpigmentation ("bronze" skin), hepatomegaly, and testicular atrophy. Laboratory studies show markedly elevated serum ferritin (>1000 ng/mL) and increased transferrin saturation (>55%).

**Which of the following genes is most likely mutated in this patient?**

A) *ATP7B* B) *HFE* C) *SERPINA1* D) *CER*

**Correct Answer: B) *HFE***

31. Myotonic Dystrophy Type 1 (DM1) and Type 2 (DM2) share several clinical features, such as myotonia, posterior subcapsular cataracts, and cardiac conduction defects. However, they exhibit distinct differences in their genetics, natural history, and pattern of muscle involvement.

**Which of the following best describes a key clinical distinction of DM2 compared to DM1?**

A) DM2 does not cause cataracts. B) DM2 is characterized by proximal muscle weakness and lacks a distinct congenital form. C) DM2 is typically associated with more severe cognitive impairment than DM1. D) DM2 has an earlier average age of onset and a more rapid progression than DM1.

**Correct Answer: B) DM2 is characterized by proximal muscle weakness and lacks a distinct congenital form.**

32. A 32-year-old male presents to the clinic with a pulsatile mass in the lateral neck and tinnitus. Imaging confirms a carotid body tumor (paraganglioma). His family history is notable for similar head and neck tumors in his father and paternal grandfather. However, his father’s sister (paternal aunt), who carries the same familial mutation, is asymptomatic and has never developed tumors. The genetic counselor explains that while the patient’s children have a 50% chance of inheriting the mutation, they are likely to develop the disease only if they inherit the pathogenic variant from their father.

**Based on this specific inheritance pattern (parent-of-origin effect) and the clinical presentation, which of the following genes is the most likely cause?**

A) *VHL* B) *RET* C) *SDHB* D) *SDHD*

Correct Answer: D

33. **A 30-year-old male presents to the medical genetics clinic for reproductive counseling. He has a clinical diagnosis of Proteus syndrome, characterized by asymmetric overgrowth of the left leg, macrodactyly, and cerebriform connective tissue nevi on the soles of his feet. Molecular analysis of affected tissue confirms a somatic mosaic pathogenic variant in the *AKT1* gene (c.49G>A). He is concerned about the risk of transmitting this condition to his future offspring.**

**Which of the following represents the approximate recurrence risk for his children?**

A) 50% B) 25% C) 10% D) < 1%

Correct Answer: D

34. **A 28-year-old female presents for genetic counseling because her brother is affected with Duchenne Muscular Dystrophy (DMD). Her mother is a confirmed obligate carrier. The consultand herself is healthy and has had no genetic testing. She has three healthy sons who show no signs of muscle weakness.**

**Based on the Bayesian risk calculation incorporating her clinical history (three unaffected sons), what is her revised (posterior) probability of being a carrier for the familial *DMD* mutation?**

A) 1/2 (50%) B) 1/5 (20%) C) 1/9 (11%) D) 1/17 (5.8%)

Correct Answer: C) 1/9 (11%)

35. A 4-year-old male is referred to the genetics clinic with a clinical diagnosis of Achondroplasia, characterized by rhizomelic short stature, macrocephaly, and trident hands. Targeted sequencing of the *FGFR3* gene reveals a novel heterozygous nonsense variant: c.150C>A (p.Cys50Ter). This variant is predicted to result in nonsense-mediated decay (NMD) and is absent from population databases.

**According to the ACMG/AMP variant interpretation guidelines (specifically regarding the PVS1 null variant criterion), which of the following is the correct classification approach for this variant *in the context of this patient's phenotype*?**

A) **PVS1 should be applied at Strong strength**, classifying the variant as **Pathogenic**, because nonsense variants are presumed to cause disease in the absence of benign data.

B) **PVS1 should be applied at Moderate strength**, classifying the variant as **Likely Pathogenic**, because *FGFR3* is a known disease gene, but this specific truncation has not been reported before.

C) **PVS1 should NOT be applied**, and the variant should be classified as a **Variant of Uncertain Significance (VUS)** for this phenotype, because the established mechanism for Achondroplasia is gain-of-function, not loss-of-function.

D) **PVS1 should be applied**, classifying the variant as **Pathogenic**, because *FGFR3* haploinsufficiency is the established mechanism for skeletal dysplasias including Achondroplasia.

**Correct Answer: C) PVS1 should NOT be applied, and the variant should be classified as a Variant of Uncertain Significance (VUS) for this phenotype, because the established mechanism for Achondroplasia is gain-of-function, not loss-of-function.**

36. A newborn female is evaluated in the nursery due to distinctive physical features. Examination reveals a white forelock of hair (poliosis) and lateral displacement of the inner canthi (dystopia canthorum), giving the appearance of widely spaced eyes. Her mother, who accompanies her, has brilliant blue eyes and reports that her hair turned completely gray in her early 20s. Genetic testing performed on cord blood confirms a heterozygous pathogenic variant in the *PAX3* gene.

**Based on the clinical presentation and the specific genetic finding, which of the following is most likely to be detected during the routine newborn screening process?**

A) Elevated immunoreactive trypsinogen (suggesting Cystic Fibrosis) B) Sensorineural hearing loss on auditory brainstem response (ABR) screening C) Elevated phenylalanine levels (suggesting Phenylketonuria) D) Congenital hypothyroidism (High TSH)

**Correct Answer: B) Sensorineural hearing loss on auditory brainstem response (ABR) screening**

37. A 16-year-old male is referred to the genetics clinic due to progressive visual difficulties, specifically "clumsiness" in low-light environments and a narrowing visual field. His medical history is significant for profound congenital sensorineural hearing loss, for which he received cochlear implants in infancy. His parents note that his motor milestones were delayed; he did not sit independently until 12 months and did not walk until 22 months of age.

**Ophthalmologic examination with fundoscopy reveals waxy pallor of the optic discs, attenuated retinal vessels, and peripheral "bone-spicule" pigment deposits.**

**Based on the combination of sensory deficits and the history of vestibular dysfunction (delayed walking), which of the following is the most likely diagnosis and the associated molecular defect?**

A) Usher Syndrome Type 2 (Defect in *USH2A*)

B) Usher Syndrome Type 1 (Defect in *MYO7A*)

C) Waardenburg Syndrome Type 1 (Defect in *PAX3*)

D) Pendred Syndrome (Defect in *SLC26A4*)

Correct Answer: B) Usher Syndrome Type 1 (Defect in *MYO7A*)

38. A 14-year-old male presents to the orthopedic clinic with a 2-month history of progressive pain and swelling in his right knee. His past medical history is significant for bilateral retinoblastoma diagnosed at 8 months of age, for which he was treated with enucleation and external beam radiation therapy.

On physical examination, there is a tender, palpable mass over the distal femur. A plain radiograph of the knee reveals a destructive lytic lesion in the metaphysis of the distal femur with a "sunburst" periosteal reaction and lifting of the periosteum (Codman triangle).

**Given the patient's history of a germline *RB1* pathogenic variant, which of the following is the most likely diagnosis?**

A) Ewing sarcoma B) Osteosarcoma C) Metastatic retinoblastoma D) Chondrosarcoma

Correct Answer: B) Osteosarcoma

39. A 26-year-old male presents to the urology clinic for a fertility evaluation after two years of unsuccessful attempts to conceive. His medical history is significant for recurrent otitis media in childhood and a chronic, daily productive cough since infancy. On physical examination, heart sounds are best appreciated on the right side of the chest (dextrocardia). A semen analysis reveals normal sperm count but profound asthenozoospermia (immotile sperm).

**Transmission electron microscopy of a respiratory mucosal biopsy from this patient would most likely reveal which of the following ultrastructural defects?**

A) Absence of the central pair of microtubules (9+0 arrangement) B) Absence of outer and/or inner dynein arms C) Defective chloride transport channels in the apical membrane D) Accumulation of misfolded alpha-1 antitrypsin protein

**Correct Answer: B) Absence of outer and/or inner dynein arms**

40. A 29-year-old couple presents for genetic counseling following amniocentesis. The mother is a known carrier of the classic Cystic Fibrosis mutation p.Phe508del. The father was screened and found to carry the 5T;TG13 variant in Intron 8 of the *CFTR* gene (with a normal coding sequence). The amniocentesis confirms that the male fetus is compound heterozygous: [p.Phe508del] ; [5T;TG13].

**Based on this specific genotype, which of the following is the most accurate counseling regarding the expected clinical phenotype?**

A) The 5T variant is always benign; the fetus is effectively a healthy carrier and will have no clinical symptoms.

B) The TG13 tract neutralizes the effect of the 5T allele; therefore, no splicing defect or disease will occur.

C) This genotype represents a "Severe/Severe" combination, and the child will inevitably have classic Cystic Fibrosis with pancreatic insufficiency and severe lung disease starting in infancy.

D) The combination of the 5T allele with a long TG tract (TG13) increases Exon 9 skipping; the male fetus is at very high risk for Congenital Bilateral Absence of the Vas Deferens (CBAVD) and may develop atypical (non-classic) Cystic Fibrosis with mild-to-moderate pulmonary symptoms.

**Correct Answer: D**

41. A 2-month-old infant is diagnosed with Spinal Muscular Atrophy (SMA) Type 1, confirmed by the homozygous deletion of *SMN1*. The neurology team decides to initiate treatment with Nusinersen (Spinraza), an intrathecal therapy designed to increase the production of functional SMN protein.

**Which of the following best describes the molecular mechanism of action of Nusinersen?**

A) It is a viral vector (AAV9) that delivers a functional copy of the *SMN1* gene to motor neurons.

B) It is an antisense oligonucleotide (ASO) that binds to *SMN2* pre-mRNA and promotes the inclusion of Exon 7.

C) It is a small molecule that inhibits the proteasomal degradation of the SMN protein.

D) It is a muscle growth factor that inhibits myostatin to prevent atrophy.

**Correct Answer: B**

42. A 14-year-old male is referred to the pediatric endocrinology clinic due to severe progressive obesity and delayed puberty. His parents report that he has difficulty seeing at night (nyctalopia) and bumps into objects frequently.

Physical examination reveals:

- BMI: >99th percentile (Centrally distributed obesity).
- Genitalia: Micropenis and small testes (Hypogonadism).
- Extremities: Scars on the ulnar side of both hands, consistent with the surgical removal of postaxial polydactyly in infancy.

Laboratory evaluation demonstrates dyslipidemia (elevated triglycerides and LDL) and insulin resistance. A renal ultrasound shows fetal lobulation and calyceal clubbing.

Based on the constellation of findings (Bardet-Biedl syndrome), the underlying pathogenic mechanism involves a defect in which of the following cellular structures?

A) Lysosome B) Primary Cilium C) Peroxisome D) Mitochondria

Correct Answer: B) Primary Cilium

43. **A 45-year-old female is diagnosed with endometrial carcinoma. Tumor tissue immunohistochemistry (IHC) reveals a loss of expression of the MLH1 and PMS2 proteins (dMMR). To rule out Lynch syndrome, further molecular analysis is performed on the tumor tissue, which identifies extensive hypermethylation of the *MLH1* promoter region.**

**Based on these tumor findings, which of the following is the most likely result expected from a germline multigene panel test performed on a peripheral blood sample?**

A) Heterozygous pathogenic variant in the *MLH1* gene (Lynch Syndrome) B) Homozygous pathogenic variant in the *MLH1* gene (Constitutional Mismatch Repair Deficiency) C) Germline *BRAF* V600E mutation D) Negative (Wild Type / Normal) genetic result (Sporadic Cancer)

Correct Answer: D) Negative (Wild Type / Normal) genetic result (Sporadic Cancer)

44. A 35-year-old male is diagnosed with Hypertrophic Cardiomyopathy (HCM) after presenting with exertional dyspnea. An echocardiogram reveals asymmetric septal hypertrophy. A comprehensive cardiomyopathy multi-gene panel identifies a heterozygous missense variant in the *MYH7* gene. This variant is classified as a "Variant of Uncertain Significance (VUS)" according to ACMG criteria.

The patient is concerned about his two asymptomatic children (ages 10 and 12) and asks if they should be tested for this specific genetic variant.

**Which of the following is the most appropriate management recommendation for the patient’s asymptomatic children?**

A) Perform targeted genetic testing for the *MYH7* VUS immediately; if negative, discharge them from cardiology follow-up. B) Do not perform genetic testing for the VUS; instead, recommend periodic clinical screening with electrocardiogram (ECG) and echocardiography. C) Treat the VUS as a benign finding and reassure the family that no further cardiac or genetic follow-up is necessary for the children. D) Perform genetic testing for the VUS; if positive, initiate prophylactic beta-blocker therapy.

**Correct Answer: B) Do not perform genetic testing for the VUS; instead, recommend periodic clinical screening with electrocardiogram (ECG) and echocardiography.**

45. A 3-year-old male is being followed in the pediatric endocrinology clinic for Prader-Willi Syndrome (PWS), confirmed by methylation analysis. He has a history of severe neonatal hypotonia but is now showing signs of increased appetite. His height is below the 3rd percentile. Physical examination reveals the characteristic facial phenotype (narrow bifrontal diameter, almond-shaped eyes) and decreased muscle mass.

The physician plans to initiate an FDA-approved therapy to not only increase linear growth but also significantly improve lean body mass, decrease fat mass, and ameliorate the characteristic facial dysmorphology. However, before starting this medication, a sleep study (polysomnography) and otolaryngology evaluation are ordered to rule out severe upper airway obstruction, due to a risk of sudden death.

**Which of the following therapeutic agents is being considered?**

A) Metformin B) Testosterone C) Somatropin (Recombinant Human Growth Hormone) D) Topiramate

**Correct Answer: C) Somatropin (Recombinant Human Growth Hormone)**

46. Two unrelated 6-year-old children are diagnosed with Cystic Fibrosis (CF) based on positive sweat chloride tests and clinical features of pulmonary disease.

- **Patient A:** Genetic testing reveals a homozygous **c.1521_1523delCTT (p.Phe508del)** mutation in the *CFTR* gene.
- **Patient B:** Genetic testing reveals a compound heterozygous genotype with **c.1652G>A (p.Gly551Asp)** and **c.350G>A (p.Arg117His)** mutations in the *CFTR* gene.

**Although both patients have the same disease (Cystic Fibrosis) and the defects are located within the same gene locus (*CFTR*), the specific DNA alterations differ. Which of the following genetic principles best describes this phenomenon?**

A) Locus heterogeneity B) Allelic heterogeneity C) Phenotypic heterogeneity (Clinical heterogeneity) D) Pleiotropy

**Correct Answer: B) Allelic heterogeneity**

47. A 34-year-old multigravida undergoes cell-free DNA (cfDNA/NIPT) screening at 11 weeks of gestation. The result is reported as "High Risk" for Trisomy 13. To confirm the diagnosis, the patient undergoes an amniocentesis at 16 weeks. The cytogenetic analysis (karyotype) of the amniocytes reveals a normal fetal chromosomal constitution (46,XX). A subsequent detailed fetal ultrasound is also unremarkable.

**Which of the following biological mechanisms is the most likely cause of the discordant results between the screening test and the diagnostic test?**

A) Low fetal fraction due to maternal obesity B) Confined Placental Mosaicism (CPM) C) Occult maternal malignancy D) Vanishing twin syndrome

**Correct Answer: B) Confined Placental Mosaicism (CPM)**

48. **A medical geneticist is evaluating a novel heterozygous missense variant in the *MYH7* gene detected in a proband with Hypertrophic Cardiomyopathy (HCM). The variant is currently classified as a Variant of Uncertain Significance (VUS) due to insufficient evidence. To resolve the classification, segregation analysis is performed on the extended family.**

**Which of the following family testing results would provide the strongest evidence AGAINST pathogenicity (supporting a Benign classification)?**

A) The variant is absent in the proband’s unaffected parents (De novo occurrence). B) The variant is present in the proband’s affected brother. C) The variant is absent in population databases like gnomAD. D) The variant is absent in an affected distant cousin who has the same clear clinical diagnosis of HCM.

Correct Answer: D) The variant is absent in an affected distant cousin who has the same clear clinical diagnosis of HCM.

49. A 24-year-old male presents with sudden, painless central vision loss in his right eye, followed by the left eye weeks later. He is diagnosed with Leber Hereditary Optic Neuropathy (LHON), confirmed by the identification of the m.11778G>A mutation in the ***MT-ND4*** gene. He is currently expecting a child with his healthy wife and asks about the risk of passing this condition to his offspring.

**Based on the inheritance pattern of this disorder, which of the following is the most accurate risk assessment for his children?**

A) 100% risk of inheriting the mutation, as mitochondria are distributed randomly. B) 50% risk, consistent with autosomal dominant inheritance with incomplete penetrance. C) 0% risk of inheriting the mutation, as mitochondrial DNA is exclusively maternally inherited. D) All daughters will be carriers, but sons will be unaffected (X-linked inheritance).

**Correct Answer: C) 0% risk of inheriting the mutation, as mitochondrial DNA is exclusively maternally inherited.**

50. **A 55-year-old male presents with progressive peripheral sensory-motor neuropathy and symptoms of heart failure. Cardiac biopsy reveals amyloid deposits composed of transthyretin protein. Genetic testing confirms a pathogenic variant in the *TTR* gene, diagnosing Hereditary Transthyretin-Mediated (hATTR) Amyloidosis. The neurologist initiates treatment with Patisiran, a lipid nanoparticle-encapsulated drug.**

**Which of the following cellular mechanisms best describes how this drug reduces the levels of the mutant protein?**

A) It acts as an antisense oligonucleotide (ASO) that binds to pre-mRNA to modulate splicing (Exon skipping/inclusion). B) It enters the nucleus and uses a viral vector to replace the defective *TTR* gene with a wild-type copy. C) It utilizes the RNA interference (RNAi) pathway, where a double-stranded siRNA loads into the RISC complex to catalytically degrade the target *TTR* mRNA in the cytoplasm. D) It is a small molecule stabilizer that binds to the TTR tetramer interface to prevent its dissociation into monomers.

**Correct Answer: C) It utilizes the RNA interference (RNAi) pathway, where a double-stranded siRNA loads into the RISC complex to catalytically degrade the target *TTR* mRNA in the cytoplasm.**

# Section 3: Expert-Authored (EA) Questions - Turkish

Bu sorular, bu çalışma için klinik genetik uzmanları tarafından özgün olarak oluşturulmuş olup test tarihi itibarıyla hiçbir yayımlanmış veri tabanında yer almamaktadır. Her soru, 4 seçenekli tek-en-iyi-cevap formatındadır. Doğru cevaplar belirtilmiştir.

1. 3 yaşındaki erkek çocuk, ciddi büyüme geriliği ve genel gelişimsel gecikme nedeniyle genetik kliniğine sevk edilmiştir. Hastanın tıbbi geçmişinde polihidramnios ve erken doğum öyküsü bulunmaktadır. Bebeklik döneminden beri, ciddi oral beslenme güçlükleri nedeniyle gastrostomi tüpü ile beslenmeye ihtiyaç duymaktadır.

Fizik muayenede kısa boy, dolgun dudaklar ve geniş ağız ile kaba yüz hatları ve seyrek, kıvırcık saçlar görülmektedir. Cilt yumuşak ve gevşektir, avuç içlerinde ve ayak tabanlarında belirgin derin kırışıklıklar bulunmaktadır. Kas-iskelet sistemi değerlendirmesinde bileklerde ve parmaklarda ulnar deviasyon görülmektedir. Yakın zamanda yapılan kardiyak değerlendirmede hipertrofik kardiyomiyopati saptanmış ve elektrokardiyogramda kaotik atriyal ritim (multifokal atriyal taşikardi) kaydedilmiştir.

**Aşağıdakilerden hangisi bu sendromla ilişkili en sık görülen malign tümördür?**

A) Nöroblastom

B) Rabdomiyosarkom

C) Hepatoblastom

D) Wilms tümörü

Doğru Cevap: B

2. Bir çift, tekrarlayan spontan abortus öyküsü nedeniyle genetik kliniğine başvurur. Her iki eşin fizik muayenesinde dikkat çekici bir bulgu yoktur. Sitogenetik analiz, kadın eşte normal 46,XX olarak raporlanmıştır. Erkek eşin karyotipi ise 45,XY,der(13;14)(q10;q10) olarak rapor edilmiştir. Çift, gelecekteki çocuklarının sağlığı konusunda endişelidir.

**Aşağıdakilerden hangisi, bu çiftin dengesiz kromozom anomalisine sahip canlı doğan bir çocuk sahibi olma riskinin yaklaşık olarak tahmin edilen değeridir?**

A) < %1

B) %10-15

C) %33

D) %50

Doğru Cevap: A

3. Bir çift, gelecekteki üreme seçenekleri hakkında danışmanlık almak için genetik kliniğine başvurur. 5 yaşındaki oğullarına Fanconi anemisi teşhisi konmuştur ve bunun *FANCA* genindeki bileşik heterozigot patojenik varyantlardan kaynaklandığı doğrulanmıştır. Ebeveynler, etkilenen oğullarına hematopoietik kök hücre donörü olabilecek başka bir çocuk sahibi olmak istemektedir. Gelecekteki kardeşlerinin uygun donör olma olasılığını sorarlar.

***FANCA* geni ve *HLA* kompleksinin bağımsız olarak segrege olduğunu varsayarsak, bir sonraki çocuklarının fenotipik olarak Fanconi anemiden etkilenmeme ve aynı zamanda etkilenen kardeşe HLA açısından tam uyumlu olma olasılığı nedir?**

A) 3/4 (75%)

B) 1/4 (25%)

C) 3/16 (18,75%)

D) 1/8 (12,5%)

Doğru Cevap: C

4. 29 yaşındaki bir kadın, gebelik öncesi danışmanlık için genetik kliniğine başvurur. Kendisi ve eşi yakında bir aile kurmayı planlamaktadır; ancak kadın, ailesinde görülen doğurganlık sorunları nedeniyle endişelidir. Kadının annesi 38, teyzesi ise 36 yaşında menopoza girmiştir. Hastanın adet döngüsü düzenlidir; ancak yakın zamanda yapılan hormon testlerinde folikül uyarıcı hormon (FSH) düzeyinin sınırda yüksek olduğu saptanmıştır.
   **Ailede erken yumurtalık yetmezliği (POI) öyküsü göz önüne alındığında, olası bir erkek çocuğunda entelektüel yetersizlik riskini değerlendirmek için aşağıdaki moleküler testlerden hangisi en uygundur?**

A) Trombofili paneli (örn. Faktör V Leiden, Protrombin)

B) *FMR1* CGG üçlü tekrar analizi

C) Periferik kandan karyotip analizi

D) Kromozomal Mikroarray Analizi (CMA)

Doğru Cevap: B

5. 22 yaşındaki bir kadın, *FBN1* genindeki patojenik heterozigot varyant ile doğrulanan Marfan sendromunun tedavisi için genetik kliniğine sevk edilmiştir. Tıbbi geçmişinde yüksek dereceli miyopi ve lens ektazisi bulunmaktadır. Fizik muayenede uzun boy, araknodaktili ve pektus ekskavatum deformitesi saptanmıştır. Yakın zamanda yapılan ekokardiyogramda önemli bir dilatasyonu gösterecek şekilde aort kökü Z skoru +3,5 olarak saptanmıştır.

Aort kökü dilatasyon hızını yavaşlatmak ve hemodinamik stresi azaltmak için kardiyolog farmakolojik tedaviye başlar. Aşağıdaki ilaç sınıflarından hangisi bu amaç için standart birinci basamak tedavi olarak kabul edilmektedir?

A) Kalsiyum kanal blokerleri

B) Beta-adrenerjik reseptör blokerleri

C) HMG-CoA redüktaz inhibitörleri (Statinler)

D) Loop diüretikler

Doğru Cevap: B) Beta-adrenerjik reseptör blokerleri

6. 42 yaşındaki erkek hasta, tekrarlayan nefrolitiyazis ile başvurmuştur. Laboratuvar değerlendirmelerinde albüminle düzeltilmiş kalsiyum düzeyi 11,2 mg/dL ve paratiroid hormonu (PTH) düzeyi yüksek bulunmuştur. Hastanın tıbbi öyküsünde 20 paket-yıl sigara içme öyküsü mevcuttur. Aile öyküsünde, babasının genç yaşta pankreas tümörü nedeniyle vefat etmesi bulunmaktadır. Yapılan moleküler testler *MEN1* geninde patojenik bir varyant olduğunu doğrulamıştır. Hastaya primer hiperparatiroidizmin tedavisi için subtotal paratiroidektomi planlanmıştır.

**Hastanın tanısı ve spesifik risk faktörleri göz önüne alındığında, agresif malignite riskini azaltmak için ameliyat sırasında aşağıdaki profilaktik prosedürlerden hangisi tartışılmalı ve uygulanmalıdır?**

A) Profilaktik transservikal timektomi

B) Profilaktik distal pankreatektomi

C) Profilaktik splenektomi

D) Profilaktik bilateral adrenalektomi

Doğru Cevap: A) Profilaktik transservikal timektomi

7. 45 yaşındaki erkek hasta, karın ultrasonunda tesadüfen saptanan bulgular nedeniyle nefroloji kliniğine sevk edilmiştir. Hastanın tıbbi öyküsünde, 30'lu yaşlarında iki kez spontan pnömotoraks geçirdiği belirtilmektedir. Fizik muayenede, yüzünde, özellikle burun ve yanak çevresinde ve boynunda çok sayıda küçük, ten rengi, kubbe şeklinde papüller görülmüştür. Daha sonra yapılan karın MRG'sinde, çapı 2 ila 3 cm arasında değişen bilateral, multifokal solid renal kitleler saptanmıştır.

**Hastanın klinik tablosu ve en olası altta yatan genetik sendrom göz önüne alındığında, aşağıdaki histolojik alt tiplerinden hangisi bu böbrek tümörleri için en karakteristik bulgudur?**

A) Berrak hücreli böbrek hücreli karsinom

B) Anjiyomiyolipom

C) Hibrit onkositik tümör (kromofobik ve onkositom özellikleri)

D) Tip 1 papiller renal hücreli karsinom

**Doğru Cevap: C) Hibrit onkositik tümör (kromofobik ve onkositom özellikleri)**

8. 34 yaşındaki bir kadın, çok sayıda trikilemom ve tiroidektomi gerektiren multinodüler guatr öyküsü nedeniyle genetik kliniğine sevk edilir. Fizik muayenesinde makrosefali (baş çevresi >98. persentil) ve oral papillomlar dikkat çekicidir. Genetik testler, Cowden sendromu ile uyumlu, *PTEN* geninde heterozigot patojenik varyant olduğunu doğrulamıştır. Test sonrası danışmanlık sırasında hasta, baş çevresi de 98. persentilin üzerinde olan, asemptomatik 7 yaşındaki oğlu hakkında soru sormuştur.

**7 yaşındaki oğlunun genetik testi ile ilgili en uygun öneri aşağıdakilerden hangisidir?**

A) Cowden sendromu öncelikle yetişkinlerde görülen bir kanser predispozisyon sendromu olduğu için test 18 yaşına kadar ertelenmelidir.

B) Tiroid kanseri takibi 7 yaşında başlaması gerektiğinden, testin şimdi yapılması önerilir.

C) Test, çocuk otizm spektrum bozukluğu veya gelişimsel gecikme belirtileri gösterirse yapılmalıdır.

D) Makrosefali varlığı tek başına klinik tanı konulmasına yeterli olduğundan test yapılmasına gerek yoktur.

Doğru Cevap: B

9. 5 yaşındaki bir erkek çocuk, boy kısalığının takibi için pediatrik genetik kliniğine sevk edilmiştir. Bebeklik döneminde akondroplazi tanısı konmuş ve bu tanı *FGFR3* genindeki heterozigot patojenik varyant (c.1138G>A) ile doğrulanmıştır. Fizik muayenede frontal çıkıntı, orta yüz geriliği, uzuvlarda rizomelik kısalma ve trifalanjiyal el saptanmıştır. Ebeveynler yeni farmakolojik seçeneklerle ilgilenmektedir.

**Aşağıdaki ajanlardan hangisi, akondroplazili pediatrik hastalarda lineer büyümeyi artırmak için spesifik olarak endike olan, ilk ve şu anda FDA tarafından onaylanmış tek tedavidir?**

A) Somatropin

B) Vosoritid

C) Pamidronat

D) Burosumab

**Doğru Cevap: B) Vosoritide**

10. Yeni doğmuş bir erkek bebek, makrozomi (doğum ağırlığı >97. persentil) ve hipoglisemi nedeniyle yenidoğan yoğun bakım ünitesinde değerlendirilmektedir. Fizik muayenede makroglossi, cerrahi müdahale gerektiren büyük bir omfalosel ve kulak memelerinde belirgin lineer katlantı saptanmıştır. Karın ultrasonunda hepatomegali ve iri böbrek saptanmıştır.

**Bu hastada tanıyı doğrulamak için en uygun *ilk* genetik test hangisidir?**

A) Kromozomal Mikroarray Analizi (CMA)

B) 11p15.5 bölgesinin metilasyon analizi (örn. MS-MLPA)

C) Karyotip analizi

D) Tüm Ekzom Dizileme (WES)

**Doğru Cevap: B**

11. 34 yaşındaki bir erkek, annesi pankreas kanserinden vefat ettikten sonra tıbbi genetik kliniğine danışmanlık için sevk edilir. Aile öyküsünde, 45 yaşında meme kanseri teşhisi konulan anne tarafındaki teyzesi ve prostat kanseri olan dayısı bulunmaktadır. Genetik testler, *BRCA2* geninde patojenik germline varyantını doğrular. Danışmanlık sırasında hasta, meme kanseri gelişme riskine ilişkin endişelerini dile getirir.

**Hastanın spesifik genetik sonucu (*BRCA2*) temel alındığında, aşağıdakilerden hangisi bu hastanın meme kanseri için yaklaşık yaşam boyu riskini temsil eder?**

A) < %0,1 (Genel popülasyon riski) B) %1 - %2 C) %6 - %8 D) %15 - %20

**Doğru Cevap: C**

12. 62 yaşında, evre III kolon kanseri olan bir erkek hasta, FOLFOX rejimi (5-Florourasil, Leucovorin ve Oksaliplatin) ile adjuvan kemoterapiye başlar. İlk infüzyondan on gün sonra, kritik durumda acil servise başvurur. Fizik muayenede şiddetli stomatit ve yaygın karın hassasiyeti saptanır. Laboratuvar sonuçları, Grade 4 nötropeni (ANC < 500/µL), trombositopeni ve sulu ishal nedeniyle şiddetli dehidratasyona bağlı akut renal hasar olduğunu gösterir. Onkolog, ilaç metabolizmasını etkileyen bir farmakogenetik bozukluktan şüphelenir.

**Aşağıdaki germline genetik varyantlarından hangisi bu hastanın şiddetli, yaşamı tehdit eden toksisitesinin en olası nedenidir?**

A) *UGT1A1* *28 alel homozigotluğu

B) *DPYD* fonksiyon kaybı varyantı

C) *KRAS* ekzon 2 mutasyonu

D) *TPMT* eksikliği

**Doğru Cevap: B) *DPYD* fonksiyon kaybı varyantı**

13. **Tekrarlayan pulmoner enfeksiyonlar ve steatore öyküsü olan 6 yaşındaki erkek çocuğa, pozitif ter testi sonucunda kistik fibrozis tanısı konur. Moleküler genetik analizde G551D (Sınıf III, Gating) mutasyonu saptanır. Çocuk göğüs hastalıkları uzmanı, bu spesifik mutasyonun patofizyolojisini hedef alan bir tedavi olan ivakaftor tedavisine başlanmasına karar verir.**

**Aşağıdakilerden hangisi bu ilacın moleküler etki mekanizmasını en iyi şekilde tanımlamaktadır?**

A) Hava yolundaki hücre dışı DNA'yı hidrolize ederek mukus viskozitesini azaltır.

B) Yanlış katlanmış CFTR proteininin bozulmasını önlemek için şaperon görevi görür ve proteinin hücre yüzeyine taşınmasını kolaylaştırır (Düzeltici).

C) Hücre yüzeyinde zaten mevcut olan CFTR proteinleri için klorür kanalının açılma olasılığını artırır (Potansiyalizatör).

D) Epitel sodyum kanalını (ENaC) inhibe ederek hava yolu yüzeyindeki sıvı hidrasyonunu artırır.

Doğru Cevap: C) Hücre yüzeyinde zaten mevcut olan CFTR proteinleri için klorür kanalının açılma olasılığını artırır (Potansiyalizatör).

14. 2 yaşındaki erkek çocuk, nöbet öyküsü nedeniyle genetik kliniğine sevk edilir. Nöbetler 6 aylıkken başlamış ve başlangıçta ateşle tetiklenmiştir. Zamanla, ateş olmadan da ortaya çıkan jeneralize tonik-klonik ve hemiklonik nöbetler gelişmiştir. Doğum öncesi ve doğum öyküsü dikkat çekici değildir. Fizik muayenede dismorfik özellikler veya nöro-kutanöz stigmata saptanmamıştır. Kraniyal MRG normal olarak rapor edilmiştir. Nöromotor gelişimi, nöbetlerin başlamasına kadar yaşına uygun olup, sonrasında hafif bir durgunluk gözlenmiştir. **"İzole genetik epilepsi" ön tanısı ile istenen panel testinde, aşağıdaki genlerden hangisinde patojenik varyant saptanma olasılığı en yüksektir?**

A) *SCN1A*

B) *TSC1*

C) *FMR1*

D) *KCNQ2*

Doğru Cevap: A

15. 3 günlük bir yenidoğan, sık tekrarlayan fokal tonik nöbetler nedeniyle yoğun bakım ünitesine yatırılır. Nöbetler arasında bebeğin nörolojik muayenesi normaldir. Aile öyküsünde, babanın bebeklik döneminde benzer nöbetler geçirdiği ve bu nöbetlerin birkaç ay içinde kendiliğinden düzeldiği belirtilmektedir. Kraniyal MRG ve metabolik taramada dikkat çekici bir bulgu yoktur. **Bu klinik tablonun (Benign Familial Neonatal Epilepsy) en olası genetik etiyolojisi aşağıdakilerden hangisidir?**

A) *KCNQ2* B) *MECP2* C) *POLG* D) *CHRNA4*

Doğru Cevap: A

16. 4 yaşındaki bir kız çocuğu, ilaca dirençli epilepsi ve gelişimsel gecikme nedeniyle değerlendirilmektedir. Nöbetlerin 1 yaşında başladığı ve ateşle tetiklendiği bildirilmiştir. Ailede epilepsi öyküsü yoktur. Genetik analizde PCDH19 geninde patojenik bir varyant saptanmıştır. **Bu genle ilişkili epilepsi sendromunun kalıtım paterni ve klinik özellikleri ile ilgili aşağıdaki ifadelerden hangisi doğrudur?**

A) X kromozomuna bağlıdır, ancak hastalık öncelikle heterozigot kızlarda görülür (erkekler etkilenmez).

B) Otozomal resesiftir ve sadece akraba evliliklerinde görülür.

C) Mitokondriyal kalıtım gösterir ve sadece anne tarafından aktarılır.

D) Y kromozomuna bağlı kalıtım gösterir ve sadece erkeklerde görülür.

Doğru Cevap: A

17. İzole epilepsi nedeniyle takip edilen bir hasta tedaviye direnç geliştirir. Wood lambası kullanılarak yapılan ayrıntılı cilt muayenesinde üç hipopigmente makül (kül yaprağı lekeleri) saptanır. Ardından yapılan kraniyal MRG'de kortikal tüberküller görülür.

**Bu hastada, aşağıdaki sinyal yollarından hangisinin inhibitör genlerinde (TSC1 veya TSC2) bir kusur olması beklenir?**

A) mTOR yolu

B) Wnt sinyal yolu

C) Sonic Hedgehog yolu

D) GABAerjik inhibisyon yolu

Doğru Cevap: A

18. Bir hasta, bebeklik döneminden itibaren absans benzeri bakış donukluğu, miyoklonik nöbetler ve genel gelişim geriliği ile başvurur. Nöbetlerin özellikle **açlık** ile tetiklendiği ve ketojenik diyete dramatik bir şekilde yanıt verdiği gözlemlenir. BOS analizi, normal kan glikozuna rağmen düşük glikoz seviyesi (hipoglikoraki) **olduğunu** ortaya koyar. **En olası genetik tanı nedir?**

A) *SLC2A1* (GLUT1 Eksikliği Sendromu)

B) *CACNA1H*

C) *SCN2A*

D) *ALDH7A1*

Doğru Cevap: A

19. 38 yaşındaki bir erkek hastaya kolorektal kanser teşhisi konmuştur. Ailesinde birkaç nesildir kolon ve endometriyal kanser vakaları görülmektedir. Germline genetik testleri, Lynch Sendromu tanısı ile uyumlu olarak MSH2 geninde patojenik heterozigot varyant olduğunu doğrulamıştır. Genetik danışmanlık seansı sırasında, hasta, "ne bekleyeceğimizi bilmek için" asemptomatik 10 yaşındaki kızının da ailesel mutasyon testi yaptırmasını talep etmiştir.

**Yetişkinlikte ortaya çıkan hastalıklar için reşit olmayanların genetik testlerine ilişkin mesleki etik kurallara (ACMG/NSGC) göre, aşağıdakilerden hangisi en uygun öneridir?**

A) Gözetim müdahaleleri genellikle erken yetişkinlik döneminde başladığından, test, kız çocuğu reşit olana (genellikle 18 yaşına ulaşana) kadar ertelenmelidir.

B) Lynch Sendromu için tarama kolonoskopisinin 10 yaşında başlaması önerildiğinden, test hemen yapılmalıdır.

C) Çocukluk döneminde tıbbi müdahale gerekmese bile, ebeveynlerin endişesini gidermek için testin şimdi yapılması önerilir.

D) Lynch sendromu çocukluk çağı lösemisi ve beyin tümörleri için de yüksek risk taşıdığından, testler hemen yapılmalıdır.

Doğru Cevap: A) Gözetim müdahaleleri genellikle erken yetişkinlik döneminde başladığından, test, kız çocuğu reşit olana (genellikle 18 yaşına ulaşana) kadar ertelenmelidir.

20. 17 yaşındaki bir kadın, rutin kan tahlillerinde açlık glikozunun 135 mg/dL ve HbA1c'nin %7,2 olduğu ortaya çıktıktan sonra endokrinoloji kliniğine sevk edilir. Kadın obez değildir ve Tip 1 diyabetle ilişkili otoantikorları yoktur. Tıbbi geçmişinde tekrarlayan idrar yolu enfeksiyonları dikkat çekicidir. Aile öyküsünde, babasının 25 yaşında diyabet teşhisi konduğu ve 30'lu yaşlarında diyaliz gerektiren son dönem böbrek hastalığı geliştirdiği belirtilmektedir.

Karın ultrasonu yapıldı ve pankreasın gövdesi ve kuyruğunda atrofiye bağlı hipoplazi saptandı. Ayrıca, bilateral renal kortikal kistler ve sol böbrekte hipoplazi tespit edildi. Laboratuvar incelemesinde de açıklanamayan, kalıcı hipomagnezemi saptandı.

Klinik tablo ve aile öyküsü göz önüne alındığında, bu hastanın durumunun en olası nedeni aşağıdaki genlerden hangisidir?

A) HNF1A (MODY 3) B) GCK (MODY 2) C) HNF1B (MODY 5) D) HNF4A (MODY 1)

Doğru Cevap: C

21. Erkek bir yenidoğan, önemli rizomelik kısalık ve mikrognati nedeniyle yenidoğan yoğun bakım ünitesinde değerlendirilmektedir. Oftalmolojik muayenede bilateral konjenital katarakt saptanır. İskelet radyografilerinde humerus ve femur epifizlerinde yaygın noktalı kalsifikasyonla ve vertebra gövdelerinde koronal yarıklar görülmektedir. Biyokimyasal analizlerde normal plazma Çok Uzun Zincirli Yağ Asidi (VLCFA) düzeyleri, ancak belirgin şekilde azalmış kırmızı kan hücresi plazmalojen düzeyleri ve yaşa bağlı fitanik asit artışı saptanmıştır.

**Klinik ve biyokimyasal bulgulara dayanarak, aşağıdakilerden hangisi en olası genetik bozukluk ve bununla ilişkili hücresel organeldir?**

A) PEX1 geni – Peroksizom (Zellweger Spektrum Bozukluğu)

B) EBP geni – Endoplazmik Retikulum (X'e bağlı Dominant Kondrodisplazi Punctata)

C) PEX7 geni – Peroksizom (Rizomelik Kondrodisplazi Punktata Tip 1)

D) ARSE geni – Golgi Aparatı (X'e bağlı Resesif Kondrodisplazi Punktata)

Doğru Cevap: C) *PEX7* geni – Peroksizom (Rizomelik Kondrodisplazi Punktata Tip 1)

22. 39 yaşındaki bir kadın, ileri anne yaşı nedeniyle gebeliğin 11. haftasında koryon villus örneklemesi (CVS) yaptırır. Sitogenetik sonuç, Trizomi 15 (47,XX,+15) olarak rapor edilir. Tanı doğrulamak için 16. haftada amniyosentez yapılır ve normal kadın karyotipi (46,XX) saptanır. Hamilelik sonuna kadar devam eder. Yenidoğan, Prader-Willi sendromunu kuvvetle düşündüren şiddetli hipotoni, beslenme bozukluğu ve hipogonadizm ile başvurur.

**Prenatal testler ile sonraki klinik tablo arasındaki tutarsızlığa dayanarak, bu spesifik sonuca yol açan fetal karyotipin normalleşmesini en iyi açıklayan hücresel mekanizma aşağıdakilerden hangisidir?**

A) Monosomi kurtarma B) Gamet tamamlama C) Trizomi kurtarma D) Döllenme sonrası mitotik ayrılmama

**Doğru Cevap: C) Trizomi kurtarma**

23. **17 yaşındaki fenotipik olarak kadın, primer amenore şikayeti ile tıbbi genetik kliniğine başvurur. Hiç adet görmemiştir. Geçmiş tıbbi öyküsü dikkat çekici değildir.**

**Fizik muayenede şunlar saptanmıştır:**

- **Boy:** 50. persentil.
- **Meme gelişimi:** Tanner evre 4 (Normal/İyi gelişmiş).
- **Kasık/Koltuk altı kılları:** Tanner Evresi 1 (seyrek veya yok).
- **Genital muayene:** Kısa, kör uçlu vajinal kese ile normal kadın dış genital organları.

**Pelvik ultrasonda rahim veya fallop tüpleri görüntülenememekte, ancak inguinal kanalda bilateral solid kitleler tespit edilmektedir. Yapılan kromozom analizinde 46,XY karyotipi saptanmıştır.**

**Aşağıdakilerden hangisi bu hastanın klinik tablosunun en olası altta yatan etiyolojisidir?**

A) *SRY* geninin silinmesine bağlı gonadal disgenez (Swyer sendromu)

B) *AR* gen mutasyonuna bağlı androjen reseptöründe bozukluk (Tam Androjen Duyarsızlık Sendromu)

C) *SRD5A2* eksikliğine bağlı testosteronun dihidrotestosterona dönüşümünde bozukluk

D) Gelişimsel bozukluğa bağlı Müllerian kanallarında agenez (Mayer-Rokitansky-Küster-Hauser sendromu)

**Doğru Cevap: B) *AR* gen mutasyonu nedeniyle androjen reseptöründe bozukluk (Tam Androjen Duyarsızlık Sendromu)**

24. **26 yaşındaki doğum sonrası bir kadın, senkopik bir olay yaşadıktan sonra kardiyoloji kliniğine başvurur. Olayın sabahın erken saatlerinde, alarm saatinin çalmasıyla aniden irkildiği sırada meydana geldiğini bildirir. Egzersiz sırasında göğüs ağrısı veya çarpıntı hissetmediğini belirtir. Aile öyküsünde, duygusal stres döneminde 30 yaşında aniden ölen bir kız kardeşi vardır. EKG'de düzeltilmiş QT aralığı (QTc) 510 ms ve düşük amplitüdlü, çentikli T dalgaları görülmektedir.**

**Spesifik tetikleyici (işitsel uyarıcı) ve EKG bulgularına göre, aşağıdaki genlerden hangisinde fonksiyon kaybına yol açan patojenik varyant en olası neden olabilir?**

A) *KCNQ1* (LQT1) B) *KCNH2* (LQT2) C) *SCN5A* (LQT3) D) *ANK2* (LQT4)

Doğru Cevap: B) *KCNH2* (LQT2)

25. **34 yaşındaki erkek hasta, ilerleyen kas sertliği ve el sıkıştıktan sonra elini gevşetmede zorluk şikayetiyle nöroloji kliniğine başvurur. Ayrıca erken başlangıçlı katarakt ve belirgin yorgunluk şikayetleri de vardır. Fizik muayenede frontal kellik, temporalde seyrek saçlar ve bilateral pitozis saptanır. Refleks çekiciyle tenar eminensine vurulduğunda, sürekli kas kasılması gözlenir. Genetik testler, *DMPK* geninin 3' UTR bölgesinde CTG trinükleotid tekrar genişlemesi (>100 tekrar) olduğunu doğrulamıştır.**

**Aşağıdaki moleküler mekanizmalardan hangisi bu hastada gözlenen miyotoninin patogenezini en iyi şekilde açıklamaktadır?**

A) CLCN1 genindeki fonksiyon kaybı mutasyonları, klorür iletkenliğinin azalmasına yol açar. B) Mutant RNA'nın toksik fonksiyon kazancı, *CLCN1* pre-mRNA'nın anormal eklemesine neden olur. C) Nöromüsküler kavşakta postsinaptik asetilkolin reseptörlerinin otoimmün yıkımı. D) Sodyum kanallarının inaktivasyonunda gecikmeye neden olan SCN4A genindeki fonksiyon kazanımı mutasyonu.

Doğru Cevap: B) Mutant RNA'nın toksik fonksiyon kazancı, *CLCN1* pre-mRNA'nın anormal eklemesine neden olur.

26. 25 yaşındaki erkek hasta, ellerinde dinlenme sırasında olan tremor, dizartri ve artan sakarlık şikayetleri ile nöroloji kliniğine başvurur. Tıbbi öyküsünde, açıklanamayan karaciğer fonksiyon testlerinde yükselme dikkat çekicidir. Oftalmolojik muayenede korneada Kayser-Fleischer halkaları saptanır.

**Aşağıdakilerden hangisi bu hasta için en olası tanı ve beklenen laboratuvar bulgusudur?**

A) Hemokromatozis – Transferrin satürasyonunda artış

B) Wilson hastalığı – Serum seruloplazmin düzeyinde azalma

C) Huntington hastalığı – Artmış CAG tekrar sayısı

D) Alkolik Karaciğer Hastalığı – Yükselmiş GGT

**Doğru Cevap:** B (Wilson Hastalığı – Serum seruloplazmin düzeyinde azalma)

27. 2 aylık bir kız bebek, konjenital anomali değerlendirmesi için tıbbi genetik kliniğine getirilir. Bebek, sol tarafta yarık dudak ve damak ile doğmuştur ve şu anda kraniyofasiyal ekip tarafından tedavi edilmektedir. Fizik muayenede, alt dudağın kırmızı kenarında iki küçük, bilateral paramedian çöküntü (sinüs) görülür. Aile öyküsünde, hastanın babasının alt dudağında benzer çöküntüler olduğu, ancak yarık öyküsü olmadığı belirtilmektedir.

**Klinik bulgular ve aile öyküsü temelinde, aşağıdakilerden hangisi en olası tanıdır?**

A) İzole (sendromik olmayan) Dudak ve Damak Yarığı

B) Van der Woude sendromu

C) 22q11.2 delesyon sendromu (DiGeorge/Velokardiyofasiyal)

D) Trizomi 13 (Patau sendromu)

**Doğru Cevap: B) Van der Woude sendromu**

28. 12 yaşındaki bir kız çocuğu, süt dişlerinin kalıcılığı ve daimi dişlerin çıkmaması nedeniyle diş hekimi tarafından genetik kliniğine sevk edilir. Panoramik diş röntgeni, çok sayıda fazla (gömülü) diş ve çıkmamış azı dişlerinin çevresinde kist oluşumu olduğunu gösterir.

Fizik muayenede hastanın hafif boy kısalığı mevcuttur. Kraniyofasiyal özellikler arasında frontal bossing, hipertelorizm ve kalıcı açık ön fontaneli gösteren palpe edilebilir yumuşak bölge bulunur. Kas-iskelet sistemi muayenesinde dar, eğimli ve aşırı hareketli omuzlar görülür, bu da hastanın omuzlarını orta hatta yaklaştırmasına olanak tanır**.**

**Bu karakteristik klinik bulgulara dayanarak, aşağıdakilerden hangisi en olası tanıdır?**

A) Osteogenezis Imperfecta

B) Piknodizostoz

C) Kleidokraniyal displazi (cleidocranial dysplasia)

D) Akondroplazi

Doğru Cevap: C) Kleidokraniyal displazi

29. **14 yaşındaki bir kız çocuğu, boy kısalığı ve ergenlik gecikmesi nedeniyle endokrinoloji kliniğine sevk edilir. Fizik muayenede yuvarlak yüz, obezite ve deri altı kemikleşmeler saptanır. El röntgeninde 4. metakarpal kemiklerde belirgin kısalık görülür (brahidaktili tip E).**

**Laboratuvar değerlendirmeleri şu sonuçları vermektedir:**

- **Kalsiyum:** Düşük
- **Fosfor:** Yüksek
- **Paratiroid Hormonu (PTH):** Yüksek
- **TSH:** Yüksek (normal serbest T4 ile)
- **FSH:** Yüksek

**Klinik fenotip (Albright Kalıtsal Osteodistrofi) ve çoklu hormon direncine ilişkin laboratuvar bulgularına göre, aşağıdaki genlerden hangisinde işlev kaybına yol açan mutasyon en olası neden olabilir?**

A) *FGFR3* B) *SHOX* C) *GNAS* D) *AIRE*

Doğru Cevap: C) GNAS

30. 48 yaşındaki erkek hasta yorgunluk, eklem ağrısı ve libido kaybı şikayetiyle başvurmuştur. Yakın zamanda diabetes mellitus tanısı konmuştur. Fizik muayenede genel hiperpigmentasyon ("bronz" cilt), hepatomegali ve testis atrofisi saptanmıştır. Laboratuvar tetkiklerinde serum ferritin düzeyinde belirgin yükselme (>1000 ng/mL) ve transferrin satürasyonunda artış (>%55) saptanmıştır.

**Bu hastada aşağıdaki genlerden hangisi mutasyona uğramış olma olasılığı en yüksektir?**

A) *ATP7B* B) *HFE* C) *SERPINA1* D) *CER*

**Doğru Cevap: B) *HFE***

31. Miyotonik Distrofi Tip 1 (DM1) ve Tip 2 (DM2), miyotoni, posterior subkapsüler katarakt ve kardiyak iletim bozuklukları gibi birçok klinik özelliği paylaşır. Ancak, genetik yapıları, doğal seyirleri ve kas tutulumlarının paternleri açısından belirgin farklılıklar gösterirler.

**Aşağıdakilerden hangisi DM2'nin DM1'e kıyasla temel klinik farkını en iyi şekilde tanımlamaktadır?**

A) DM2 kataraktlara neden olmaz. B) DM2, proksimal kas güçsüzlüğü ile karakterizedir ve belirgin bir konjenital formu yoktur. C) DM2, tipik olarak DM1'den daha şiddetli kognitif bozukluklarla ilişkilidir. D) DM2, DM1'den daha erken bir ortalama başlangıç yaşına ve daha hızlı bir ilerlemeye sahiptir.

**Doğru Cevap: B) DM2, proksimal kas güçsüzlüğü ile karakterizedir ve belirgin bir konjenital formu yoktur.**

32. 32 yaşındaki erkek hasta, boyun yan tarafında nabız atan bir kitle ve kulak çınlaması şikayeti ile kliniğe başvurur. Görüntüleme sonuçları karotis cisimciği tümörü (paraganglioma) olduğunu doğrular. Aile öyküsünde babasında ve babasının babasında benzer baş ve boyun tümörleri görülmüştür. Ancak, aynı ailesel mutasyonu taşıyan halası asemptomatiktir ve hiç tümör gelişmemiştir. Genetik danışmada, hastanın çocuklarının mutasyonun kalıtılma olasılığının %50 olduğunu, ancak babalarından patojenik varyantı almaları halinde hastalığa yakalanma olasılıklarının yüksek olduğunu açıklamıştır.

**Bu spesifik kalıtım paterni (köken ebeveyn etkisi) ve klinik tablo göz önüne alındığında, aşağıdaki genlerden hangisi en olası neden olabilir?**

A) *VHL* B) *RET* C) *SDHB* D) *SDHD*

Doğru Cevap: D

33. **30 yaşındaki bir erkek, üreme danışmanlığı için tıbbi genetik kliniğine başvurur. Sol bacağında asimetrik aşırı büyüme, makrodaktili ve ayak tabanlarında serebriform bağ dokusu nevüsleri ile karakterize Proteus sendromu klinik tanısı vardır. Etkilenen dokunun moleküler analizi, AKT1 geninde (c.49G>A) somatik mozaik patojenik varyant olduğunu doğrular. Bu durumun gelecekteki çocuklarına geçme riskinden endişe duymaktadır.**

**Aşağıdakilerden hangisi, çocukları için yaklaşık tekrarlama riskini temsil eder?**

A) %50 B) %25 C) %10 D) < %1

Doğru Cevap: D

34. **28 yaşındaki bir kadın, erkek kardeşi Duchenne Musküler Distrofi (DMD) hastası olduğu için genetik danışmanlık almaya gelmiştir. Annesi, zorunlu taşıyıcı olduğu doğrulanmıştır. Danışan kadın sağlıklıdır ve genetik test yaptırmamıştır. Kas güçsüzlüğü belirtisi göstermeyen üç sağlıklı oğlu vardır.**

**Klinik öyküsünü (etkilenmemiş üç oğlu) içeren Bayes risk hesaplamasına göre, ailesel *DMD* mutasyonunun taşıyıcısı olma olasılığı (posterior) ne kadardır?**

A) 1/2 (50%) B) 1/5 (20%) C) 1/9 (11%) D) 1/17 (5,8%)

Doğru Cevap: C) 1/9 (11%)

35. 4 yaşındaki bir erkek çocuk, rizomelik kısa boy, makrosefali ve trifalangeal el ile karakterize olan akondroplazi klinik tanısı ile genetik kliniğine sevk edilmiştir. FGFR3 geninin hedefli dizilemesi, yeni bir heterozigot nonsense varyantı ortaya çıkarmıştır: c.150C>A (p.Cys50Ter). Bu varyantın nonsense aracılı bozunma (NMD) ile sonuçlanacağı tahmin edilmektedir ve popülasyon veritabanlarında bulunmamaktadır.

**ACMG/AMP varyant yorumlama kılavuzlarına göre (özellikle PVS1 null varyant kriteri ile ilgili olarak), *bu hastanın fenotipi bağlamında* bu varyant için doğru sınıflandırma yaklaşımı aşağıdakilerden hangisidir?**

A) **PVS1**, **güçlü bir şekilde uygulanmalı** ve varyantı **patojenik** olarak sınıflandırmalıdır, çünkü saçma varyantların benign verilerin yokluğunda hastalığa neden olduğu varsayılır.

B) *FGFR3* bilinen bir hastalık geni olmasına rağmen, bu spesifik kesiklik daha önce bildirilmemiştir, bu nedenle **PVS1 Orta güçte uygulanmalı** ve varyant **Muhtemelen Patojenik** olarak sınıflandırılmalıdır.

C) **PVS1 uygulanmamalı** ve varyant, bu fenotip için **Klinik önemi Belirsiz (VUS)** olarak sınıflandırılmalıdır, çünkü akondroplazi için belirlenmiş mekanizma fonksiyon kaybı değil, fonksiyon kazancıdır.

D) **PVS1 uygulanmalı** ve varyant **Patojenik** olarak sınıflandırılmalıdır, çünkü *FGFR3* haploinsufficiency, Akondroplazi dahil iskelet displazileri için bilinen mekanizmadır.

**Doğru Cevap: C) PVS1 uygulanmamalı ve varyant, bu fenotip için Klinik önemi Belirsiz (VUS) olarak sınıflandırılmalıdır, çünkü Akondroplazi için belirlenmiş mekanizma fonksiyon kaybı değil, fonksiyon kazancıdır.**

36. Yeni doğmuş bir kız bebek, belirgin fiziksel özellikleri nedeniyle bebek odasında değerlendirilir. Muayenede, beyaz bir saç tutamı (poliozis) ve iç göz köşelerinin yana doğru kayması (distopia canthorum) saptanır, bu da gözlerin birbirinden uzak görünmesine neden olur. Ona eşlik eden annesi parlak mavi gözlere sahiptir ve 20'li yaşlarının başında saçlarının tamamen griye döndüğünü bildirir. Kordon kanından yapılan genetik testler, PAX3 geninde heterozigot patojenik bir varyant olduğunu doğrulamaktadır.

**Klinik tablo ve spesifik genetik bulgulara dayanarak, rutin yenidoğan tarama sürecinde aşağıdakilerden hangisi en olası şekilde tespit edilir?**

A) Yüksek immünoreaktif tripsinojen (kistik fibrozis şüphesi) B) Sensörinöral işitme kaybı (işitsel beyin sapı yanıtı sonucuna bakınız) C) Yüksek fenilalanin düzeyleri (fenilketonüri şüphesi) D) Konjenital hipotiroidizm (yüksek TSH)

**Doğru Cevap: B) Sensörinöral işitme kaybı (Auditory Brainstem Response sonucuna bakınız)**

37. 16 yaşındaki erkek hasta, ilerleyen görme sorunları, özellikle düşük ışık ortamlarında "sakarlık" ve daralan görme alanı nedeniyle genetik kliniğine sevk edilmiştir. Tıbbi geçmişinde, bebeklik döneminde koklear implant takılan derin konjenital sensörinöral işitme kaybı bulunmaktadır. Ailesi, motor gelişiminde gecikme olduğunu belirtmiştir; 12 aylık olana kadar tek başına oturamamış ve 22 aylık olana kadar yürüyememiştir.

**Fundoskopi ile yapılan oftalmolojik muayenede optik disklerde mumsu solukluk, zayıflamış retina damarları ve periferik "kemik iğnesi" pigment birikintileri görülmüştür.**

**Duyusal bozukluklar ve vestibüler disfonksiyon öyküsü (yürüme gecikmesi) kombinasyonuna göre, aşağıdakilerden hangisi en olası tanı ve ilişkili moleküler bozukluktur?**

A) Usher Sendromu Tip 2 (*USH2A*'da defekt)

B) Usher Sendromu Tip 1 (*MYO7A*'da defekt)

C) Waardenburg Sendromu Tip 1 (*PAX3*'te defekt)

D) Pendred Sendromu (*SLC26A4*'te defekt)

Doğru Cevap: B) Usher Sendromu Tip 1 (*MYO7A'da* defekt)

38. 14 yaşındaki erkek hasta, 2 aydır sağ dizinde ilerleyen ağrı ve şişlik şikayeti ile ortopedi kliniğine başvurur. Hastanın tıbbi öyküsünde, 8 aylıkken teşhis edilen bilateral retinoblastom ve bunun tedavisi için enükleasyon ve harici ışın radyoterapi uygulanması önemlidir.

Fizik muayenede, distal femur üzerinde hassas, palpe edilebilir bir kitle vardır. Dizin düz radyografisinde, distal femurun metafizinde "güneş ışını" periosteal reaksiyon ve periosteumun kalkması (Codman üçgeni) ile birlikte yıkıcı litik lezyon görülmektedir.

**Hastanın germ hattı *RB1* patojenik varyantı öyküsü göz önüne alındığında, aşağıdakilerden hangisi en olası tanıdır?**

A) Ewing sarkomu B) Osteosarkom C) Metastatik retinoblastom D) Kondrosarkom

Doğru Cevap: B) Osteosarkom

39. 26 yaşındaki erkek hasta, iki yıldır başarısız gebelik denemelerinin ardından üreme değerlendirmesi için üroloji kliniğine başvurur. Tıbbi öyküsünde çocukluk çağında tekrarlayan orta kulak iltihabı ve bebeklikten beri günlük kronik balgamlı öksürük öyküsü vardır. Fizik muayenede, kalp sesleri göğsün sağ tarafında daha belirgindir (dextrocardia). Sperm analizi normal sperm sayısını ancak ciddi asthenozoospermi (hareketsiz sperm) olduğunu gösterir.

**Bu hastadan alınan solunum mukozası biyopsisinin transmisyon elektron mikroskobu incelemesi, aşağıdaki ultrastrüktürel bozukluklardan hangisini ortaya çıkaracaktır?**

A) Merkezi mikrotübül çiftinin yokluğu (9+0 düzeni) B) Dış ve/veya iç dinein kollarının yokluğu C) Apikal membranda kusurlu klorür taşıma kanalları D) Yanlış katlanmış alfa-1 antitripsin proteininin birikimi

**Doğru Cevap: B) Dış ve/veya iç dinein kollarının yokluğu**

40. 29 yaşındaki bir çift, amniyosentez sonrası genetik danışmanlık için başvurur. Anne, klasik kistik fibroz mutasyonu p.Phe508del taşıyıcısı olduğu bilinmektedir. Baba taramadan geçirilmiş ve *CFTR* geninin intron 8'inde (normal kodlama dizisi ile) 5T;TG13 varyantı taşıdığı tespit edilmiştir. Amniyosentez, erkek fetüsün bileşik heterozigot olduğunu doğrulamıştır: [p.Phe508del] ; [5T;TG13].

**Bu spesifik genotipe dayanarak, beklenen klinik fenotip ile ilgili en doğru danışmanlık aşağıdakilerden hangisidir?**

A) 5T varyantı her zaman iyi huyludur; fetüs aslında sağlıklı bir taşıyıcıdır ve klinik semptomları olmayacaktır.

B) TG13 bölgesi, 5T alelinin etkisini nötralize eder; bu nedenle, kırpılma kusuru veya hastalık meydana gelmez.

C) Bu genotip "Şiddetli/Şiddetli" bir kombinasyonu temsil eder ve çocuk kaçınılmaz olarak bebeklik döneminde başlayan pankreas yetmezliği ve şiddetli akciğer hastalığı ile klasik Kistik Fibrozis'e sahip olacaktır.

D) 5T alelinin uzun TG tekrarı (TG13) ile kombinasyonu, Ekzon 9 atlamasını artırır; erkek fetüs, Konjenital Bilateral Vas Deferens Yokluğu (CBAVD) riski çok yüksektir ve hafif ila orta derecede pulmoner semptomlarla atipik (klasik olmayan) Kistik Fibrozis geliştirebilir.

**Doğru Cevap: D**

41. 2 aylık bir bebekte, *SMN1'in* homozigot delesyonu ile doğrulanan Tip 1 Spinal Musküler Atrofi (SMA) teşhisi konur. Nöroloji ekibi, fonksiyonel SMN proteini üretimini artırmak için tasarlanmış bir intratekal tedavi olan Nusinersen (Spinraza) ile tedaviye başlamaya karar verir.

**Aşağıdakilerden hangisi Nusinersen'in moleküler etki mekanizmasını en iyi şekilde tanımlamaktadır?**

A) *SM*N1 geninin fonksiyonel bir kopyasını motor nöronlara taşıyan viral bir vektördür (AAV9).

B) *SMN2* pre-mRNA'ya bağlanan ve Ekson 7'nin dahil edilmesini destekleyen bir antisens oligonükleotiddir (ASO).

C) SMN proteininin proteazomal bozunmasını inhibe eden küçük bir moleküldür.

D) Atrofiye engel olmak için miyostatini inhibe eden bir kas büyüme faktörüdür.

**Doğru Cevap: B**

42. 14 yaşındaki bir erkek çocuk, şiddetli ilerleyici obezite ve gecikmiş ergenlik nedeniyle pediatrik endokrinoloji kliniğine sevk edilir. Ailesi, çocuğun geceleri görme zorluğu (nyctalopia) yaşadığını ve sık sık nesnelere çarptığını bildirir.

Fizik muayenede şunlar saptanır:

- BMI: >99. persentil (Merkezi dağılımlı obezite).
- Genital organlar: Mikropenis ve küçük testisler (Hipogonadizm).
- Ekstremiteler: Her iki elin ulnar tarafında, bebeklik döneminde postaksiyal polidaktili cerrahi olarak çıkarılmasına uygun yara izleri.

Laboratuvar değerlendirmeleri dislipidemi (yüksek trigliserid ve LDL) ve insülin direnci olduğunu göstermektedir. Böbrek ultrasonunda fetal lobülasyon ve kaliks klubing görülmektedir.

Bulguların bütünlüğüne (Bardet-Biedl sendromu) dayanarak, altta yatan patojenik mekanizma aşağıdaki hücresel yapılardan hangisinde bir kusuru içermektedir?

A) Lizozom B) Primer Silium C) Peroksizom D) Mitokondri

Doğru Cevap: B) Primer Silium

43. **45 yaşındaki bir kadına endometrial karsinom teşhisi konmuştur. Tümör dokusunun immünohistokimyasal (IHC) incelemesinde MLH1 ve PMS2 proteinlerinin ekspresyon kaybı (dMMR) saptanmıştır. Lynch sendromunu ekarte etmek için tümör dokusu üzerinde ileri moleküler analizler yapılmış ve *MLH1* promotör bölgesinde yaygın hipermetilasyon saptanmıştır.**

**Bu tümör bulgularına dayanarak, periferik kan örneğinde yapılan germline multigen panel testinden beklenen en olası sonuç aşağıdakilerden hangisidir?**

A) MLH1 geninde heterozigot patojenik varyant (Lynch sendromu) B) MLH1 geninde homozigot patojenik varyant (konstitüsyonel uyumsuzluk onarım eksikliği) C) Germ hattı *BRAF* V600E mutasyonu D) Negatif (Yabanıl tip/normal) genetik sonuç (sporadik kanser)

Doğru Cevap: D) Negatif (Yabanıl Tip / Normal) genetik sonuç (Sporadik Kanser)

44. 35 yaşındaki bir erkek, efor dispnesi şikayeti ile başvurduktan sonra hipertrofik kardiyomiyopati (HCM) tanısı alır. Ekokardiyogramda asimetrik septal hipertrofi saptanır. Kapsamlı bir kardiyomiyopati çoklu gen paneli, MYH7 geninde heterozigot bir missense varyantı tespit eder. Bu varyant, ACMG kriterlerine göre "Klinik önemi belirsiz Varyant (VUS)" olarak sınıflandırılır.

Hasta, semptomsuz iki çocuğu (10 ve 12 yaşında) hakkında endişelidir ve bu spesifik genetik varyant için test yaptırmaları gerekip gerekmediğini sorar.

**Aşağıdakilerden hangisi, hastanın asemptomatik çocukları için en uygun tedavi önerisidir?**

A) *MYH7* VUS için hemen hedefli genetik test yapın; negatif çıkarsa, kardiyoloji takibinden çıkarın. B) VUS için genetik test yapmayın; bunun yerine, elektrokardiyogram (EKG) ve ekokardiyografi ile periyodik klinik tarama önerin. C) VUS'u iyi huylu bir bulgu olarak değerlendirin ve aileye çocukların kardiyak veya genetik takip gerektirmediğini söyleyin. D) VUS için genetik test yapın; pozitif çıkarsa, profilaktik beta bloker tedavisi başlatın.

**Doğru Cevap: B) VUS için genetik test yapmayın; bunun yerine, elektrokardiyogram (EKG) ve ekokardiyografi ile periyodik klinik tarama yapılmasını önerin.**

45. 3 yaşındaki bir erkek çocuk, metilasyon analizi ile doğrulanan Prader-Willi Sendromu (PWS) nedeniyle pediatrik endokrinoloji kliniğinde takip edilmektedir. Ağır neonatal hipotoni öyküsü vardır, ancak şu anda iştah artışı belirtileri göstermektedir. Boyu 3. persentilin altındadır. Fizik muayenede karakteristik yüz fenotipi (dar bifrontal çap, badem şekilli gözler) ve azalmış kas kütlesi saptanmıştır.

Doktor, sadece boy uzamasını artırmakla kalmayıp, aynı zamanda yağsız vücut kütlesini önemli ölçüde artırmak, yağ kütlesini azaltmak ve karakteristik yüz dismorfolojisini iyileştirmek için FDA onaylı bir tedaviye başlamayı planlamaktadır. Ancak, bu ilacı başlatmadan önce, ani ölüm riski nedeniyle şiddetli üst solunum yolu tıkanıklığını dışlamak için bir uyku çalışması (polisomnografi) ve kulak burun boğaz değerlendirmesi istenmiştir.

**Aşağıdaki tedavi ajanlarından hangisi düşünülmektedir?**

A) Metformin B) Testosteron C) Somatropin (Rekombinant İnsan Büyüme Hormonu) D) Topiramat

**Doğru Cevap: C) Somatropin (Rekombinant İnsan Büyüme Hormonu)**

46. İki akraba olmayan 6 yaşındaki çocuğa, pozitif ter klorür testleri ve pulmoner hastalığın klinik özellikleri temelinde kistik fibroz (CF) tanısı konmuştur.

- **Hasta A:** Genetik testler, *CFTR* geninde homozigot **c.1521_1523delCTT (p.Phe508del)** mutasyonu olduğunu ortaya koymaktadır.
- **Hasta B:** Genetik testler, CFTR geninde **c.1652G>A (p.Gly551Asp)** ve **c.350G>A (p.Arg117His)** mutasyonları ile bileşik heterozigot genotip ortaya çıkarmıştır.

**Her iki hasta da aynı hastalığa (kistik fibroz) sahip olmasına ve defekt aynı gen lokusunda (*CFTR*) bulunmasına rağmen, spesifik DNA değişiklikleri farklıdır. Aşağıdaki genetik ilkelerden hangisi bu fenomeni en iyi şekilde tanımlamaktadır?**

A) Lokus heterojenliği B) Alel heterojenliği C) Fenotipik heterojenlik (Klinik heterojenlik) D) Pleiotropi

**Doğru Cevap: B) Alel heterojenliği**

47. 34 yaşındaki multigravida bir kadın, gebeliğinin 11. haftasında hücre dışı DNA (cfDNA/NIPT) taramasına girer. Sonuç, Trizomi 13 için "Yüksek Risk" olarak rapor edilir. Tanıyı doğrulamak için, hasta 16. haftada amniyosentez yapılır. Amniyositlerin sitogenetik analizi (karyotip) normal fetal kromozom yapısını (46,XX) ortaya koyar. Ardından yapılan ayrıntılı fetal ultrason da dikkat çekici bir bulgu göstermez.

**Aşağıdaki biyolojik mekanizmalardan hangisi, tarama testi ile tanı testi arasındaki uyumsuz sonuçların en olası nedenidir?**

A) Anne obezitesi nedeniyle düşük fetal fraksiyon B) Plasentaya Sınırlı Mozaiklik (Confined Placental Mosaicism, CPM) C) Gizli anne malignitesi D) Kaybolan ikiz sendromu

**Doğru Cevap: B) Plasentaya Sınırlı Mozaiklik (Confined Placental Mosaicism, CPM)**

48. **Bir tıbbi genetikçi, hipertrofik kardiyomiyopati (HCM) olan bir probandda tespit edilen MYH7 genindeki yeni bir heterozigot missense varyantını değerlendirmektedir. Varyant, yetersiz kanıt nedeniyle şu anda Klinik önemi belirsiz (VUS) olarak sınıflandırılmaktadır. Sınıflandırmayı çözmek için, geniş aile üzerinde segregasyon analizi yapılır.**

**Aşağıdaki aile test sonuçlarından hangisi patojeniteye KARŞI en güçlü kanıtı sağlar (benign (iyi huylu) sınıflandırmayı destekler)?**

A) Varyant, probanda etkilenen ebeveynlerde bulunmamaktadır (De novo oluşumu). B) Varyant, probanda etkilenen erkek kardeşte bulunmaktadır. C) Varyant, gnomAD gibi popülasyon veritabanlarında bulunmamaktadır. D) Varyant, aynı net klinik HCM tanısı olan etkilenen uzak bir kuzeninde bulunmamaktadır.

Doğru Cevap: D) Varyant, aynı net klinik HCM tanısı olan etkilenen uzak kuzeninde bulunmamaktadır.

49. 24 yaşındaki erkek hasta, sağ gözünde ani, ağrısız merkezi görme kaybı ile başvurur, birkaç hafta sonra sol gözünde de aynı durum görülür. MT-ND4 geninde m.11778G>A mutasyonu tespit edilerek Leber Kalıtsal Optik Nöropati (LHON) tanısı konur. Hasta, sağlıklı eşi ile bir çocuk beklemektedir ve bu hastalığın çocuğuna geçme riskini sorar.

**Bu hastalığın kalıtım şekline göre, çocukları için en doğru risk değerlendirmesi aşağıdakilerden hangisidir?**

A) Mitokondri rastgele dağıldığı için mutasyonu miras alma riski %100'dür. B) Tamamlanmamış penetranslı otozomal dominant kalıtımla tutarlı olarak %50 risk. C) Mitokondriyal DNA sadece anne tarafından kalıtıldığı için mutasyonu kalıtma riski %0. D) Tüm kız çocukları taşıyıcı olacak, ancak erkek çocukları etkilenmeyecek (X'e bağlı kalıtım).

**Doğru Cevap: C) Mitokondriyal DNA sadece anne tarafından miras alındığı için mutasyonu miras alma riski %0'dır.**

50. **55 yaşındaki erkek hasta, ilerleyici periferik duyusal-motor nöropati ve kalp yetmezliği semptomları ile başvurur. Kardiyak biyopsi, transtiretin proteininden oluşan amiloid birikintileri ortaya çıkarır. Genetik testler, TTR geninde patojenik bir varyant olduğunu doğrular ve Kalıtsal Transtiretin Aracılı (hATTR) Amiloidoz tanısı konur. Nörolog, lipit nanopartikül kapsüllü bir ilaç olan Patisiran ile tedaviye başlar.**

**Aşağıdaki hücresel mekanizmalardan hangisi bu ilacın mutant protein düzeylerini nasıl azalttığını en iyi şekilde açıklamaktadır?**

A) Splicing'i (ekzon atlama/dahil etme) modüle etmek için pre-mRNA'ya bağlanan bir antisens oligonükleotid (ASO) olarak işlev görür. B) Çekirdeğe girer ve viral bir vektör kullanarak kusurlu *TTR* genini vahşi tip bir kopya ile değiştirir. C) RNA interferans (RNAi) yolunu kullanır; burada çift sarmallı siRNA, RISC kompleksine yüklenerek sitoplazmada hedef *TTR* mRNA'yı katalitik olarak parçalar. D) TTR tetramer arayüzüne bağlanarak monomerlere ayrışmasını önleyen küçük moleküllü bir stabilizatördür.

**Doğru Cevap: C) RNA interferans (RNAi) yolunu kullanır; burada çift sarmallı siRNA, RISC kompleksine yüklenerek sitoplazmada hedef *TTR* mRNA'yı katalitik olarak parçalar.**

# Section 4: Bilingual Item-Matching Table and Answer Key (EA Items)

The table below provides the definitive one-to-one correspondence between the English and Turkish versions of the 50 expert-authored (EA) items, together with the answer key. Identically numbered items refer to the same question in both languages. The 50 published board-review (PBR) items were administered in the same order (PBR 1–50) in both languages; their topics are listed in Section 1 and their answer keys follow the source textbook.

| **Item** | **English item (opening words)** | **Turkish item (opening words)** | **Key** |
| --- | --- | --- | --- |
| 1 | A 3-year-old male is referred to the genetics clinic… | 3 yaşındaki erkek çocuk, ciddi büyüme geriliği ve genel… | B |
| 2 | A couple presents to the genetics clinic due to… | Bir çift, tekrarlayan spontan abortus öyküsü nedeniyle genetik kliniğine… | A |
| 3 | A couple presents to the genetics clinic for counseling… | Bir çift, gelecekteki üreme seçenekleri hakkında danışmanlık almak için… | C |
| 4 | A 29-year-old female presents to the genetics clinic for… | 29 yaşındaki bir kadın, gebelik öncesi danışmanlık için genetik… | B |
| 5 | A 22-year-old female is referred to the genetics clinic… | 22 yaşındaki bir kadın, FBN1 genindeki patojenik heterozigot varyant… | B |
| 6 | A 42-year-old male presents with recurrent nephrolithiasis. Laboratory evaluation… | 42 yaşındaki erkek hasta, tekrarlayan nefrolitiyazis ile başvurmuştur. Laboratuvar… | A |
| 7 | A 45-year-old male is referred to the nephrology clinic… | 45 yaşındaki erkek hasta, karın ultrasonunda tesadüfen saptanan bulgular… | C |
| 8 | A 34-year-old female is referred to the genetics clinic… | 34 yaşındaki bir kadın, çok sayıda trikilemom ve tiroidektomi… | B |
| 9 | A 5-year-old male is referred to the pediatric genetics… | 5 yaşındaki bir erkek çocuk, boy kısalığının takibi için… | B |
| 10 | A newborn male is evaluated in the neonatal intensive… | Yeni doğmuş bir erkek bebek, makrozomi (doğum ağırlığı >97.… | B |
| 11 | A 34-year-old male is referred to the medical genetics… | 34 yaşındaki bir erkek, annesi pankreas kanserinden vefat ettikten… | C |
| 12 | A 62-year-old male with Stage III colon cancer initiates… | 62 yaşında, evre III kolon kanseri olan bir erkek… | B |
| 13 | A 6-year-old male with a history of recurrent pulmonary… | Tekrarlayan pulmoner enfeksiyonlar ve steatore öyküsü olan 6 yaşındaki… | C |
| 14 | A 2-year-old male is referred to the genetics clinic… | 2 yaşındaki erkek çocuk, nöbet öyküsü nedeniyle genetik kliniğine… | A |
| 15 | A 3-day-old neonate is admitted to the intensive care… | 3 günlük bir yenidoğan, sık tekrarlayan fokal tonik nöbetler… | A |
| 16 | A 4-year-old female is evaluated for drug-resistant epilepsy and… | 4 yaşındaki bir kız çocuğu, ilaca dirençli epilepsi ve… | A |
| 17 | A patient followed for isolated epilepsy develops resistance to… | İzole epilepsi nedeniyle takip edilen bir hasta tedaviye direnç… | A |
| 18 | A patient presents with absence-like staring spells, myoclonic seizures,… | Bir hasta, bebeklik döneminden itibaren absans benzeri bakış donukluğu,… | A |
| 19 | A 38-year-old male is diagnosed with colorectal cancer. His… | 38 yaşındaki bir erkek hastaya kolorektal kanser teşhisi konmuştur.… | A |
| 20 | A 17-year-old female is referred to the endocrinology clinic… | 17 yaşındaki bir kadın, rutin kan tahlillerinde açlık glikozunun… | C |
| 21 | A male newborn is evaluated in the neonatal intensive… | Erkek bir yenidoğan, önemli rizomelik kısalık ve mikrognati nedeniyle… | C |
| 22 | A 39-year-old female undergoes Chorionic Villus Sampling (CVS) at… | 39 yaşındaki bir kadın, ileri anne yaşı nedeniyle gebeliğin… | C |
| 23 | A 17-year-old phenotypic female presents to the clinic with… | 17 yaşındaki fenotipik olarak kadın, primer amenore şikayeti ile… | B |
| 24 | A 26-year-old postpartum female presents to the cardiology clinic… | 26 yaşındaki doğum sonrası bir kadın, senkopik bir olay… | B |
| 25 | A 34-year-old male presents to the neurology clinic with… | 34 yaşındaki erkek hasta, ilerleyen kas sertliği ve el… | B |
| 26 | A 25-year-old male presents to the neurology clinic with… | 25 yaşındaki erkek hasta, ellerinde dinlenme sırasında olan tremor,… | B |
| 27 | A 2-month-old female infant is brought to the genetics… | 2 aylık bir kız bebek, konjenital anomali değerlendirmesi için… | B |
| 28 | A 12-year-old female is referred to the genetics clinic… | 12 yaşındaki bir kız çocuğu, süt dişlerinin kalıcılığı ve… | C |
| 29 | A 14-year-old female is referred to the endocrinology clinic… | 14 yaşındaki bir kız çocuğu, boy kısalığı ve ergenlik… | C |
| 30 | A 48-year-old male presents with fatigue, arthralgia, and loss… | 48 yaşındaki erkek hasta yorgunluk, eklem ağrısı ve libido… | B |
| 31 | Myotonic Dystrophy Type 1 (DM1) and Type 2 (DM2)… | Miyotonik Distrofi Tip 1 (DM1) ve Tip 2 (DM2),… | B |
| 32 | A 32-year-old male presents to the clinic with a… | 32 yaşındaki erkek hasta, boyun yan tarafında nabız atan… | D |
| 33 | A 30-year-old male presents to the medical genetics clinic… | 30 yaşındaki bir erkek, üreme danışmanlığı için tıbbi genetik… | D |
| 34 | A 28-year-old female presents for genetic counseling because her… | 28 yaşındaki bir kadın, erkek kardeşi Duchenne Musküler Distrofi… | C |
| 35 | A 4-year-old male is referred to the genetics clinic… | 4 yaşındaki bir erkek çocuk, rizomelik kısa boy, makrosefali… | C |
| 36 | A newborn female is evaluated in the nursery due… | Yeni doğmuş bir kız bebek, belirgin fiziksel özellikleri nedeniyle… | B |
| 37 | A 16-year-old male is referred to the genetics clinic… | 16 yaşındaki erkek hasta, ilerleyen görme sorunları, özellikle düşük… | B |
| 38 | A 14-year-old male presents to the orthopedic clinic with… | 14 yaşındaki erkek hasta, 2 aydır sağ dizinde ilerleyen… | B |
| 39 | A 26-year-old male presents to the urology clinic for… | 26 yaşındaki erkek hasta, iki yıldır başarısız gebelik denemelerinin… | B |
| 40 | A 29-year-old couple presents for genetic counseling following amniocentesis.… | 29 yaşındaki bir çift, amniyosentez sonrası genetik danışmanlık için… | D |
| 41 | A 2-month-old infant is diagnosed with Spinal Muscular Atrophy… | 2 aylık bir bebekte, SMN1'in homozigot delesyonu ile doğrulanan… | B |
| 42 | A 14-year-old male is referred to the pediatric endocrinology… | 14 yaşındaki bir erkek çocuk, şiddetli ilerleyici obezite ve… | B |
| 43 | A 45-year-old female is diagnosed with endometrial carcinoma. Tumor… | 45 yaşındaki bir kadına endometrial karsinom teşhisi konmuştur. Tümör… | D |
| 44 | A 35-year-old male is diagnosed with Hypertrophic Cardiomyopathy (HCM)… | 35 yaşındaki bir erkek, efor dispnesi şikayeti ile başvurduktan… | B |
| 45 | A 3-year-old male is being followed in the pediatric… | 3 yaşındaki bir erkek çocuk, metilasyon analizi ile doğrulanan… | C |
| 46 | Two unrelated 6-year-old children are diagnosed with Cystic Fibrosis… | İki akraba olmayan 6 yaşındaki çocuğa, pozitif ter klorür… | B |
| 47 | A 34-year-old multigravida undergoes cell-free DNA (cfDNA/NIPT) screening at… | 34 yaşındaki multigravida bir kadın, gebeliğinin 11. haftasında hücre… | B |
| 48 | A medical geneticist is evaluating a novel heterozygous missense… | Bir tıbbi genetikçi, hipertrofik kardiyomiyopati (HCM) olan bir probandda… | D |
| 49 | A 24-year-old male presents with sudden, painless central vision… | 24 yaşındaki erkek hasta, sağ gözünde ani, ağrısız merkezi… | C |
| 50 | A 55-year-old male presents with progressive peripheral sensory-motor neuropathy… | 55 yaşındaki erkek hasta, ilerleyici periferik duyusal-motor nöropati ve… | C |

# Section 5: Model Testing Details

Each model was accessed in a fresh conversation session with default settings, apart from the response-mode selections listed below: where an interface offered more than one user-selectable response mode or configuration under the same model name, the reasoning-enabled (“thinking”) configuration was selected for all administrations. Web browsing, search, tool use, memory, and file-upload features were disabled where user-facing controls permitted, and no system or custom instructions were applied. The English and Turkish administrations were conducted in separate sessions.

| **Model** | **Developer** | **Access interface** | **Displayed model designation** | **Testing date(s)** | **Settings / tools** |
| --- | --- | --- | --- | --- | --- |
| GPT-5.2 | OpenAI | ChatGPT web application | GPT-5.2 | February 17–18, 2026 | “Thinking” mode selected (not “Auto” or “Instant”); browsing, tools, memory, and file upload disabled |
| Gemini 3 Pro | Google | Gemini web application | Gemini 3 Pro | February 17–18, 2026 | “Pro” reasoning-model selection (previously labeled “Thinking with 3 Pro”); browsing, tools, memory, and file upload disabled |
| Claude Sonnet 4.6 | Anthropic | Claude web application | Claude Sonnet 4.6 | February 17–18, 2026 | Extended-thinking toggle enabled; browsing, tools, memory, and file upload disabled |
| Grok 4 | xAI | Grok web application | Grok 4 | February 17–18, 2026 | “Expert” mode selected (not “Auto” or “Fast”); browsing, tools, memory, and file upload disabled |
| DeepSeek-V3.2 | DeepSeek | DeepSeek web application | DeepSeek-V3.2 | February 17–18, 2026 | “DeepThink” toggle enabled; browsing, tools, memory, and file upload disabled |
